# Differential Item Functioning Analysis of Likert Scales: An Overview and Demonstration of Rating Scale Tree Model

**DOI:** 10.1177/00332941241308806

**Published:** 2025-01-06

**Authors:** Farshad Effatpanah, Hamdollah Ravand, Philipp Doebler

**Affiliations:** Research Unit of Psychological Assessment, Faculty of Rehabilitation Sciences, 14311 TU Dortmund University, Dortmund, Germany; Department of English, 114738 Vali-e-Asr University of Rafsanjan, Rafsanjan, Iran; Department of Statistics, 14311 TU Dortmund University, Dortmund, Germany

**Keywords:** Likert-type scales, differential item functioning, rating scale tree model, cognitive test anxiety, gender, age

## Abstract

An important psychometric property in educational and psychological testing is differential item functioning (DIF), assessing whether different subgroups respond differently to particular items within a scale, despite having the same overall ability level. In fact, DIF occurs when respondents with the same underlying trait level have different probabilities of selecting specific response categories, depending on their subgroup membership. This study aims to demonstrate the usefulness of rating scale tree (RStree) model in detecting DIF of Likert-type scales across age and gender in social sciences. Compared to the conventional DIF methods, a priori specification of groups for detecting DIF is not required in the RStree model. The study used item responses of 721 English as a foreign language (EFL) students to a cognitive test anxiety scale. The analysis of the RStree model generated three non-predefined nodes, with slight variations in item difficulties. Four items of the scale were flagged as DIF items. Results showed that age does not have any impact on the performance of respondents, whereas gender has a role in generating DIF in test anxiety. The findings also indicated the effectiveness of the RStree model in reflecting the underlying interaction between the covariates and the scale items.

## Introduction

Likert-type scales, developed in 1932 by Renis Likert, are a commonly used standardized psychometric tool in social sciences research. They are used to operationalize and measure respondents’ perceptions, attitudes, behaviors, and knowledge. A Likert scale typically comprises a stem or a statement (e.g., After taking a test/exam, I worry that I gave the wrong answers.) and three to nine response categories or options (e.g., ‘strongly disagree’, ‘disagree’, ‘agree’, and ‘strongly agree’). Respondents should read each statement and rate the extent to which they agree or disagree with that particular statement. Responses are usually assigned numerical values for analysis, where higher numbers indicate stronger agreement or endorsement of the statement. A higher level of the trait is signified by agreement with positively worded statements, whereas responses to negatively phrased statements are reverse scored. The scores on all of the items are summed to generate an overall scale score, which is interpreted as reflecting the level of the expected trait (Baghaei & Effatpanah, 2024). Because Likert scales are used to capture the variation and complexity of respondents’ attitudes as well as making different research claims, it is essential to check psychometric characteristics of a scale and validate score interpretations.

Researchers often investigate the psychometric properties of an entire instrument, including Likert scales, by employing statistical methods such as reliability analysis, factor analysis, and confirmatory factor analysis (CFA) within classical test theory (CTT), as well as rating scale analysis and item fit analysis within item response theory (IRT). For example, Cronbach’s alpha can be used to assess reliability, while rating scale models, many of which are extensions of the Rasch model (Rasch, 1960/1980), help examine item functioning in Likert-type scales. An important psychometric property in educational and psychological testing is measurement invariance which holds when the measurement properties of a test remain consistent across different subgroups (e.g., age, gender, race/ethnicity) and under varying circumstances, such as different testing conditions, time points, or contexts of administration (Engelhard, 2014). Measurement invariance is crucial for ensuring the validity and comparability of measurements across various subgroups, time points, or conditions. In the context of Likert scales, this can be achieved through multi-group CFA or IRT-based models. These methods assess whether it is plausible that the same latent construct is being measured across groups by evaluating equality constraints of parameters such as factor loadings, item thresholds, and intercepts. Note that the group-specific distributions of the latent variable are not constrained, so differences in latent mean and variance are meaningful when measurement invariance holds. This allows researchers to interpret and make meaningful comparisons about group differences without potential invalidation by measurement biases. Failure to find measurement invariance suggests that the scale may not be appropriate for use across different subgroups or that observed differences reflect true disparities in the latent trait being measured, rather than measurement error (Maassen et al., 2023).

Measurement invariance at the item level is typically assessed by differential item functioning (DIF), sometimes referred to as item bias. DIF analysis evaluates whether different subgroups respond differently to specific items within a scale by comparing the probabilities of a correct response or selecting response categories across groups at equivalent levels of the underlying trait. In the absence of DIF, all differences in response behavior at the group level are due to group-level differences in the distribution of the latent variables. When DIF is present, it suggests that the item functions differently for different subgroups, indicating that the item may not measure the same construct consistently across those groups. This lack of measurement invariance can jeopardize the validity of test score interpretations and uses, as it implies that observed differences in scores may be influenced by group membership rather than true differences in the trait being measured (American Educational Research Association [AERA] et al., 2014). A variety of methods, which will be reviewed in the following section, have been developed to detect DIF. Although these methods have high statistical power for DIF detection, they face several major limitations (Strobl et al., 2015). First, a common problem with them is that two or more groups (the so-called focal and reference groups), typically determined by researchers, should be specified prior to analyzing DIF. Second, in most conventional methods, numeric variables, such as age, are usually categorized before testing, leading to the loss of valuable information. Additionally, there is “a lack of cognitive theories backing the choice of grouping variables (covariates), which has resulted in the proliferation of DIF analysis across [covariates] such as gender, age, or first language, to name a few” (Aryadoust, 2018, p. 197).

Along the same lines, conventional DIF detection methods, such as the Mantel-Haenszel (Holland & Thayer, 1988) method, logistic regression (Swaminathan & Rogers, 2000), and IRT-based analyses (Raju, 1988; Steinberg & Thissen, 2006), have been widely used in different fields to investigate DIF across groups and often yield significant insights into identifying DIF caused by various variables. The Mantel-Haenszel method compares the performance of focal and reference groups on specific items while controlling for total score to determine if item response patterns differ. Logistic regression, on the other hand, examines the probability of responding to an item correctly based on group membership while controlling for ability levels. IRT models, such as the Rasch model, assess differences in item parameters to evaluate whether items function equivalently across groups. However, the assumption with using these conventional methods is that members within a specific subgroup share homogeneous characteristics (Russell et al., 2022). Whether implicitly or explicitly, the methods presume that members within the same group exhibit similar response patterns, thereby allowing for accurate generalizations from the group level to subgroup levels (Aryadoust et al., 2024). Nevertheless, several researchers have indicated the presence of heterogeneity within subgroups (Russell et al., 2022). For instance, studies have shown that DIF items identified as favoring a particular gender group are inclined to benefit only a segment of the members within that gender group, rather than the entirety of the group’s members (Grover & Ercikan, 2017; Oliveri et al., 2014). Disregarding this variability within subgroups can induce inaccurate identification and interpretation of DIF, potentially resulting in unfair and biased tests (Knott et al., 2017). Therefore, more robust statistical methods are required to explore heterogeneity within subgroups and split them and generate further subgroups with homogeneous response patterns.

As a new psychometric technique, Komboz et al. (2018) recently introduced rating scale tree (RStree) and partial credit tree (PCtree) models for detecting DIF in polytomous items. Henninger et al. (2024, Preprint) expanded upon these models by incorporating an effect size measure for DIF and differential step functioning (DSF) in items scored on a polytomous scale. This enhancement improves the interpretability of results concerning tree size, identification of DIF and DSF items, and quantification of effect sizes. The advantage of the models over the conventional methods is that no priori specification of focal and reference groups is required, continuous variables can be used, and heterogeneity within subgroups can be identified. Despite the effectiveness of the RStree model in detecting DIF, too little attention has been paid to its application in social sciences.

Against this background, the present study aims to demonstrate the usefulness of the RStree model for investigating DIF of polytomous items in social sciences using item responses of 721 English as a foreign language (EFL) students to a cognitive test anxiety scale (Cassady & Finch, 2014; Cassady & Johnson, 2002). More specifically, this study seeks to show whether the model can capture the interaction between covariates causing DIF in Likert scales.

## Background

### Cognitive Test Anxiety

Over the past few decades, there has increasingly been a research interest in studying test anxiety among educational and psychological researchers. Although there is still a lack of consensus over its definition, Zeidner (1998) defined test anxiety as a set of “phenomeno-logical, physiological, and behavioral responses that accompany concern about possible negative consequences or failure on an exam or similar evaluative situation” (p. 17). The traditional model for test anxiety considered this construct unidimensional whose manifestation was displayed by self-deprecating rumination and physiological arousal (Mandler & Sarason, 1952; Sarason, 1984). However, further examinations into test anxiety among students unveiled the presence of at least two separate dimensions. Liebert and Morris (1967) proposed a bifactor model and showed that test anxiety comprises both worry and emotionality as distinct and subordinate factors. The ‘emotionality’ factor (also known as physiological or affective) mostly focuses on physiological signs of stress, such as headache and dry mouth, whereas the ‘worry’ component concerns self-critical rumination, intrusive thoughts, and other cognitive distractions specifically related to testing contexts (Zeidner, 1998). Additionally, numerous researchers differentiated between trait-like and state-like anxiety in testing situations (Zohar, 1998) and emphasized the impact of test anxiety beyond the testing event itself (Cassady, 2004; Schwarzer & Jerusalem, 1992).

Several well-established psychometric scales have been developed for measuring test anxiety (e.g., Benson et al., 1992; Cassady & Johnson, 2002; Rost & Schermer, 2007; Spielberger, 1980). Among them, the Cognitive Test Anxiety Scale (CTAS) developed by Cassady and Johnson (2002) is one of the most widely used scales for measuring cognitive test anxiety in research across cultural contexts. Research on the CTAS has shown that its development over the past two decades has led to an adaptive and valid measure of the cognitive aspects of test anxiety. This scale effectively differentiates various levels of cognitive test anxiety, making it a reliable tool for both research and practical applications (Cassady, 2023). Drawing on previous research that examined the nature of test anxiety and identified worry and emotionality as two important dimensions of this construct, the scale was developed. The scale was specifically designed to examine the extensive range of indicators associated with worry throughout all stages of the learning and testing process (Cassady & Johnson, 2002). The decision to concentrate on cognitive aspects, rather than emotionality, stemmed from Hembree’s (1988) meta-analysis and related studies, highlighting that the cognitive dimension (i.e., “worry”) exerted the most significant negative effect on performance.

The CTAS is a 27-item self-report measure of the cognitive domain of test anxiety, with high internal consistency (Cronbach’s alpha = 0.91) and strong validity evidence through comparisons with the Reactions to Tests scale (Sarason, 1984) and the Tension and Worry subscales (Cassady & Johnson, 2002). During the scale validation process for the Argentina translation (Furlan et al., 2009), however, analyses revealed that the use of reverse-coded items on the original CTAS produced a second factor that had already been unidentified in the original scale validation process. The findings of Furlan et al. (2009) showed that the reverse-coded items loaded onto a separate factor, interpreted as assessing “test confidence” rather than a “low level of test anxiety”. By removing all reverse-coded items, Cassady and Finch (2014) proposed a short single-factor version of the CTAS comprising 17 items (CTAS-17). The CTAS and its revised versions have been translated into a number of languages, such as Chinese (Chen, 2007; Zheng, 2010), German (Stefan et al., 2020), Persian (Baghaei & Cassady, 2014), Spanish (Andujar & Cruz-Martínez, 2020; Araujo Torrejón & Moreno Martinez, 2021; Furlan et al., 2009), and Turkish (Bozkurt et al., 2017). For a comprehensive review on the development of the CTAS and its versions, refer to Cassady (2023).

Along the same lines, research has indicated that the demographic characteristics of respondents, such as age and gender, have the potential to influence their levels of test anxiety, though research in this area is still inconclusive (Torrano et al., 2020). Regarding age, research has shown the impact of age on test anxiety. Older respondents have been found to exhibit higher levels of test anxiety compared to their younger counterparts (e.g., Nwosu et al., 2022). Furthermore, previous studies on identifying the prevalence of cognitive test anxiety have routinely reported significant differences in test anxiety between females and males, with females tending to have higher levels of anxiety at all levels of education than males (e.g., Bandalos et al., 1995; Cassady & Johnson, 2002; Putwain, 2007; Torrano et al., 2020; Zeidner, 1990). However, Wen et al. (2020) found that males are likely to experience higher levels of test anxiety than females.

Several researchers have also specifically examined the DIF of the CTAS and its versions across gender. Some researchers reported gender equivalence for item responses (e.g., Bozkurt et al., 2017), while other studies showed that some items differentially function across females and males (e.g., Baghaei & Cassady, 2014). For example, in Bozkurt et al.’s (2017) study, using a hybrid, iterative, ordinal logistic regression method, the DIF analysis indicated that the items of the scale performed equivalently for both genders in the sample, although some observed gender differences were observed. The differences in scores likely reflected a generally higher level of cognitive test anxiety among high school females in Turkey compared to their male counterparts. Additionally, as reported by Baghaei and Cassady (2014), the analysis of the Persian version of the CTAS-17’s construct stability across genders indicated that Items 1 and 8 exhibited differential functioning, with male students finding these items significantly easier to endorse. Their results diverged with general findings in test anxiety research, which typically show higher anxiety levels in females (e.g., Cassady, 2023; Zeidner, 1998). The examination of DIF, however, differed from studies that had compared overall anxiety levels between males and females, as it focused on individual item response patterns. In their sample, the response differences on these two specific items did not indicate that males experience higher levels of test anxiety. Instead, the data suggested that males in the Iranian sample were more inclined to endorse items related to perseverating on thoughts of test failure. The authors proposed two possible explanations for this pattern: (1) most males participated in their study were engineering majors, a field associated with high-stakes testing and higher anxiety levels, while most females studied humanities, which generally involves less exam pressure; and (2) societal expectations related to job security may place greater pressure on males, as failure for them could delay graduation and reduce job prospects, while for females, failure might simply mean repeating a course without the same career-related consequences. Controversial findings of previous studies could point to the need for further studies to investigate DIF of the CTAS and the impact of several covariates on the performance of respondents.

### Differential Item Functioning

DIF occurs when items on a scale work differently for or against a particular group, such as females and males (Zumbo, 2007). In fact, an item is labeled as exhibiting DIF when respondents with the same underlying latent trait level but from different groups have a different probability of getting an item right or endorsing a response category. In the context of test anxiety, individuals with the same levels of anxiety might display varying response patterns due to factors unrelated to anxiety itself. This type of DIF can result in biased scores and lead to misinterpretations of test outcomes. There are a variety of methods, available in the literature, which can be utilized to analyze DIF. The methods can be classified into two general groups. The first group refers to total score methods which use a matching variable as a criterion for splitting the sample into different groups, known as reference and focal groups. Examples of such methods include Scheuneman’s chi-square (Scheuneman, 1979), Camilli’s chi-square (Ironson, 1982), the Mantel-Haenszel test procedure (Holland & Thayer, 1988), the generalized Mantel-Haenszel (Fidalgo & Madeira, 2008; Zwick et al., 1993), and logistic regression modeling and its extensions (De Boeck & Wilson, 2004; Swaminathan & Rogers, 2000; Tay et al., 2011; Van den Noortgate & De Boeck, 2005).

The second group refers to Rasch (Rasch, 1960/1980)/IRT modeling (Magis et al., 2015) which assumes that an IRT model holds within each group. The approach entails the comparison of the item characteristic curves (ICCs) between two or more pre-specified groups (Linn et al., 1981) or the comparison of the item parameters across them. To compare item parameters, various techniques are utilized including the likelihood ratio (LR) test (Andersen, 1973), Lord’s chi square test (Lord, 1980), the generalized Lord Test (Kim et al., 1995), and Raju method (Raju, 1988). There are also further test statistics suggested by Holland and Wainer (1993), Thissen et al. (1993), and Woods et al. (2013).

The advantage of the two approaches is that they provide a clear interpretation of results when certain items are labeled as DIF. They help in discerning which items present greater difficulty for specific groups of respondents, thereby offering valuable insights into formulating hypotheses concerning the psychological sources of DIF and devising strategies to eliminate or prevent it in future test versions (Strobl et al., 2015). However, a major problem with these methods is the requirement to specify two or more groups before conducting the DIF analysis. Researchers must explicitly define the groups (e.g., gender or age) they wish to compare, which can lead to a narrow focus and potentially overlook other relevant subgroup characteristics. For example, if a study focuses solely on comparing males and females, it might miss detecting DIF related to other meaningful variables like age, socioeconomic status, or cultural background. As a result, this pre-specification could introduce an artificial difference between groups that might not truly exist in a more nuanced or thorough analysis. In fact, there exists a risk that any detected DIF may be an artifact of the predefined group comparison rather than a genuine item-level difference across subgroups (Komboz et al., 2018). Additionally, although some DIF detection methods such as the ordinal (or binary) logistic regression approach (Crane et al., 2006) and Multiple Cause Multiple Indicator models (Jöreskog & Goldberger, 1975) can be used to evaluate DIF on a continuous predictor (e.g., age), most conventional DIF methods require the categorization of continuous or numeric variables into discrete groups before conducting DIF analysis. This categorization can result in a loss of valuable information because it oversimplifies the data. For example, when age is split into arbitrary categories like “young” and “old,” subtle differences in age-related effects like (inverse) U-shaped are lost. Individuals within a broad age group may have differing response patterns, and this categorization masks those subtleties, reducing the precision of the analysis. In this regard, important variations that exist within each age group are ignored, leading to less informative and potentially misleading results (Strobl et al., 2015).

Another method for detecting DIF in the Rasch model is the latent class (or mixture distribution) approach, or typically called mixture Rasch model (Rost, 1990), which integrates latent class analysis (Lazarsfeld & Henry, 1968) and Rasch modelling. In this model, the sample is divided into several latent classes, which groups individuals based on their response patterns to test items. The logic behind the model is that while the Rasch model holds within each latent class, the ordering of item difficulties may vary across these classes. Specifically, the model assumes that individuals within each latent class share similar item response behaviors, while item parameter estimates, such as item difficulty, can vary across these latent classes. This differentiation allows for the identification of latent subgroups that respond differently, even if they have the same overall ability level (Rost, 1990). Several covariates, such as age, gender, or educational background, can then be used to explore the qualitative differences between the latent classes. These covariates help researchers understand how certain characteristics might influence membership in a particular latent class, shedding light on the distinct features of each group (Effatpanah et al., 2024a, 2024b). For more elaboration on this approach, we refer the interested reader to De Boeck and Wilson (2004).

### Dichotomous and Polytomous Rasch Models

IRT (also referred to as Modern Test Theory) is a mathematical framework aimed at quantifying latent traits based on the fundamental premise that a person’s response to an item is a function of the difference between his/her abilities and the characteristics of the item. Within the framework, the Rasch model (Rasch, 1960/1980) stands out by considering difficulty or facility as the primary parameter for evaluating items. The model was conceptualized in the 1950s by the Danish mathematician and statistician Georg Rasch for evaluating achievement tests among school children. Beyond its initial application in educational assessments, the Rasch model has been widely used in the social sciences for the analysis of data from tests and scales in education and psychology. It has recently garnered attention in clinical and public health research, serving as a valuable tool for exploring a broad range of health-related phenomena, such as rehabilitation and community violence.

The Rasch model is a probabilistic model utilized to predict the performance of persons on several test items. The assumption of the model is that the probability of getting an item right or endorsing a response category is a function of the difficulty level of the item and the ability of a person. The greater a person’s ability relative to an item difficulty, the higher is the probability of success or endorsement of a higher category. For the standard Rasch model, the item response function is defined as:

(1) $$P\left(X_{vj}=1\;|\;\theta_{v},\beta_{j}\;\right)=\frac{\exp\left(\theta_{v}-{\;\beta }_{j}\right)\;}{1+\exp\left(\theta_{v}-{\;\beta }_{j}\right)}$$

where $P\left(X_{vj}\right)$ denotes the probability of solving item j for respondent v ; $X_{vj}$ shows the response of person ν to item j ; $\theta_{v}$ is the ability of person ν ; and $\beta_{j}$ is the difficulty of the item j .

Based on the Rasch model, Andrich (1978) developed the rating scale model (RSM), also called the polytomous Rasch model, as an extension of the dichotomous Rasch model for the analysis of responses to items with ordered categories or Likert-type scales. For the RSM, the response functions are explained as:

(2) $$P\left(X_{vj}=x_{vj}\;|\;\theta_{v},\beta_{j},\tau \;\right)=\frac{\exp\overset{x_{vj}}{\underset{k=0}{\sum }}\;\left(\theta_{v}-\left(\beta_{j}+\tau_{k}\right)\right)}{\overset{m}{\underset{l=0}{\sum }}\exp\;\overset{l}{\underset{\;k=0}{\sum }}\;\left(\theta_{v}-\left(\beta_{i}+\tau_{k}\right)\right)}$$

where $P\left(X_{vj}\right)$ denotes the probability of endorsing item j with m categories $\left(x_{vj}\;\epsilon \;\left\{0,1,2,\ldots ,m\right\}\right)$ ; $\theta_{\nu }$ is the ability of person ν ; $\beta_{i}$ is the difficulty of the item j ; and $\tau_{j}$ is the rating scale category threshold. In the RSM, the threshold is the location on the latent trait continuum where the probability of marking category k and category k−1 is equal. For a rating scale consisting of m response categories, there are m−1 rating scale category thresholds.

The RSM is appropriate for modeling scales where all items share a similar structural response format (i.e., rating scale structure). Just as the dichotomous Rasch model, the RSM provides estimates of item locations and person locations on a log-odds scale that indicates the latent trait. The model also estimates a set of category thresholds for all the items that represent the difficulty associated with each pair of adjacent categories in the scale. The main assumption of the model is that there exists a series of ordered thresholds that separate the ordered categories from one another (Wind & Hua, 2022). All items have unique location parameters, but the differences between the response categories and the mean of the threshold locations are equal or uniform across all items. In other words, all items are equally discriminating, and that scoring of the response categories are equally spaced. The model allows a stringent test of the hypothesis that thresholds or response categories represent increasing levels of a latent trait.

Shortly after, Masters (1982) proposed the partial credit model (PCM), also referred to as the adjacent category logit model, as another extension of the Rasch model. For the PCM, the person-item interaction is modeled as:

(3) $$P\left(X_{vj}=x_{vj}\;|\;\theta_{v},\delta_{j}\right)=\frac{\exp\;\overset{x_{vj}}{\underset{k=0}{\sum }}\;\left(\theta_{v}-\delta_{jk}\right)}{\overset{m_{j}}{\underset{l=0}{\sum }}\exp\;\overset{l}{\underset{k=0}{\sum }}\;\left(\theta_{v}-\delta_{jk}\right)}$$

where $\delta_{j}$ shows threshold parameter for item j .

The PCM, like the RSM, is well-suited for analyzing instruments that involve polytomous items comprising numerous ordered categories, including items found in attitude questionnaires, aptitude or achievement tests, and performance assessments. Similar to the RSM, the PCM provides estimates of item locations, person locations, and rating scale category thresholds on a log-odds scale that indicates the latent trait. It also assumes an equal discrimination across all items among respondents. However, the PCM includes two main assumptions which differentiate it from the RSM. First, the number of response categories can vary across items; some may be on a 4-point scale, others on a 5-point scale, and some may even be dichotomous. Therefore, separate rating scale category thresholds for each item included in the analysis is estimated. Second, “[the] PCM does not require the thresholds to follow the same order as the response categories. Because PCM considers adjacent categories in each step, the adjacent response categories are treated as a series of dichotomous items, but without order constraints beyond adjacent categories” (Desjardins & Bulut, 2018, p. 145). If an item comprises only two categories, the PCM simplifies to the Rasch model. The RSM is commonly regarded as a constrained version of the PCM.

### Rating Scale Tree Model

With respect to the limitations of the conventional DIF detection methods, Strobl et al. (2015) introduced a new method for detecting DIF in the Rasch model on the basis of a statistical algorithm known as model-based recursive partitioning (Migliorati et al., 2023; Zeileis et al., 2008). The recursively partitioning Rasch trees approach, also shortly called Rasch tree, conflates the principles of the Rasch model with recursive partitioning techniques from machine learning and econometrics. The model-based recursive partitioning framework (Debelak & Strobl, 2019) is an extension of the classification and regression tree (CART) method (Hothorn et al., 2006) and is a machine learning technique used to generate decision trees. This approach systematically divides a dataset into subsamples by iteratively splitting it based on predictor variable values, with the goal of creating homogeneous subgroups within each subsample. Individuals within these subgroups are relatively similar in terms of the outcome variable, whereas there are significant differences in the outcome variable between the subsamples (For a comprehensive review on recursive partitioning, see Strobl et al., 2009). In the model-based recursive partitioning framework (e.g., the Rasch tree), decision trees are integrated with parametric models such as the Rasch model, RSM, and PCM. Instead of partitioning the dataset to detect differences in the outcome variable, the tree structure now identifies splits based on model parameters, including location or threshold parameters in the RSM (Komboz et al., 2018).

The Rasch tree has the capability to distinguish various subgroups of individuals who display diverse response patterns across a set of given items or tasks. The approach involves iteratively examining all conceivable groups formed by combinations of existing covariates, which maintains the interpretability of the results while thoroughly identifying a broad range of potential DIF indicators (Strobl et al., 2015). Compared to the conventional DIF detection methods, the Rasch tree does not require prior specification of group structure or the specific way covariates are associated with DIF.

Komboz et al. (2018) introduced RStree and PCtree models as elaboration of the Rasch tree to encompass polytomously scored items. They contend that this extended model confers a dual benefit. Firstly, it is capable of uncovering previously unnoticed groups of individuals exhibiting DIF. Secondly, it introduces a flexible method that can detect violations of measurement invariance at each step, called “differential step functioning” (DSF; Penfield, 2007). DSF occurs when the conditional probability of responses to specific categories varies between groups (Penfield, 2007). As noted by Komboz et al. (2018, p. 159),

by means of a sequence of binary splits, model-based recursive partitioning methods can capture any number of categories and approximate any functional shape in a data-driven way. This makes them more flexible than previous approaches and offers a methodological advantage especially for the detection of violations of measurement invariance that should not go unnoticed because a wrong group structure or functional form was assumed in the statistical test.

The process of inferring the RStree and PCtree structure involves five consecutive steps. First, one joint Rasch model should be first fit to the entire sample. Second, conditional maximum likelihood (CML) approach is used to jointly estimate model parameters for all subjects across the entire sample. Since the raw scores of persons ( $r_{v}=\overset{m}{\underset{j=1}{\sum }}X_{vj}$ ) serve as sufficient statistics for the person parameters in the Rasch model family, the item and threshold parameters can be estimated through iterative procedures based on the CML approach $L_{c}$ :

(4) $$L_{c}\left(\beta ,\tau \;|r_{1},\ldots ,r_{n}\right)=\overset{n}{\underset{v=1}{\prod }}L_{c}\left(\beta ,\tau \;|{\;r}_{v}\right)=\overset{n}{\underset{v=1}{\prod }}\frac{\exp\;\left(-\overset{m}{\underset{j=1}{\sum }}\left(X_{vj}\;.\;\beta_{j}+\sum_{k=0}^{X_{vj}}\tau_{k}\right)\right)}{\gamma_{r_{v}\;}\left(\beta ,\tau \right)}$$

(5) $$L_{c}\left(\delta \;|r_{1},\ldots ,r_{n}\right)=\overset{n}{\underset{v=1}{\prod }}L_{c}\left(\delta \;|{\;r}_{v}\right)=\overset{n}{\underset{v=1}{\prod }}\frac{\exp\;\left(-\overset{m}{\underset{j=1}{\sum }}\sum_{k=0}^{X_{vj}}\delta_{jk}\right)}{\gamma_{r_{v}\;}\left(\delta \right)}$$

In the equations, $\gamma_{r_{v}\;}$ shows the elementary symmetric functions of order $r_{v}$ (Strobl et al., 2015). The first threshold parameter of the first item is set to zero, that is, $\beta_{1}=0$ and $\tau_{1}=0$ for the RSM, and $\delta_{11}=0$ for the PCM.

Third, the stability of item or threshold parameters concerning each existing covariate is examined. Drawing upon the methodology of structural change tests commonly employed in econometrics, the consistency of model parameters across subgroups defined by covariates is tested. The tree tests all available covariates at each split, but the different splits are performed recursively. After jointly estimating model parameters for the entire sample, individual deviations (or the individual score contributions) from the joint model are ordered based on a covariate. If a systematic DIF or DSF is present regarding subgroups created by the covariate, the ordering will reveal systematic changes in individual divergences. However, in the absence of DIF or DSF, values will exhibit only random fluctuations. The covariate inducing DIF will display the highest level of instability among all covariates, and taking its instability into account will significantly enhance the model’s fit. According to Komboz et al. (2018, p. 138), instability refers to “deviation of person parameters from the overall mean zero, as estimated by the Rasch model”. To assess the statistical significance of the divergence of the parameters from the overall mean, generalized M-fluctuation tests (Zeileis et al., 2008; Zeileis & Hornik, 2007) are utilized. For each covariate, a test statistic and corresponding Bonferroni-adjusted *p*-values are computed; Maximum Lagrange-multiplier (LM) or score test statistic is used to estimate test statistics for categorical covariates, and an extended form of LM is used for continuous covariates (Zeileis et al., 2008).

Fourth, in cases of significant instability, the sample is divided based on the covariate exhibiting the most significant instability and at the cut-point that maximizes the enhancement of the model fit. More specifically, following the selection of a covariate for partitioning, the most appropriate cut-point is specified by maximizing the split log-likelihood (i.e., the sum of the log-likelihood for two distinct models). All potential cut-points are examined to pinpoint the optimal value for splitting the sample (Komboz et al., 2018). The LM test is employed to assess the presence of significant DIF or DSF within a covariate, while the LR test is utilized to estimate where the most pronounced DIF or DSF takes place (Komboz et al., 2018).

Finally, steps 2–4 are recursively repeated within the emerging subsamples to produce additional subsamples across the available covariates and alleviate all instabilities. This process continues until two stopping criteria are fulfilled: (1) when there is no more significant instability remaining across item difficulty parameters concerning the levels of any of the covariates, as indicated by a *p*-value lower than 0.05; and (2) when a minimum sample size is set for each node, determining at which point the algorithm stops splitting. Strobl et al. (2009) pointed out that the researcher can specify the minimum size based on the characteristics of the sample.

Although the RStree and PCtree models are effective in detecting DIF and DSF, they have faced two major criticisms. First, these models perform only a global invariance test to identify relevant covariates and optimal cut-points (Henninger et al., 2023). They do not assess DIF or DSF at the individual item level (Berger & Tutz, 2016). While the models determine which covariates and cut-points lead to differences in item parameters across subgroups, they do not automatically flag specific items with DIF or measure the degree of DIF for each item. The second limitation of the models is their tendency to detect minor discrepancies in item parameters, especially in larger samples, since statistical significance tests guide the decision on whether and where to make splits. This issue is common in large scale international assessments, such as the National Assessment of Educational Progress (NAEP; e.g., Johnson & Carlson, 1994), the Programme for International Student Assessment (PISA; OECD, 2022), and the Trends in International Mathematics and Science Study (TIMSS; e.g., Ferraro & Van de Kerckhove, 2006). As a result, the models often produce more partitions, creating larger decision trees and uncovering additional subgroups in larger datasets due to its increased statistical power. This expansion can make it harder for users to identify relevant covariates and DIF items, complicating the interpretation of DIF effects. Moreover, minor DIF effects, while statistically significant, may lack practical relevance (Henninger et al., 2023; Szepannek & Holt, 2024).

To resolve these issues, Henninger et al. (2024, Preprint) incorporated the partial gamma $\left(p_{\gamma }\right)$ coefficient (Barbiero & Hitaj, 2020; Bjorner et al., 1998) as an effect size measure for detecting DIF and DSF in polytomously scored items within the RStree and PCtree models. The authors also examined and implemented a correction for item wise testing to avoid the risk of falsely identifying DIF and DSF items, particularly in longer tests. Such enhancements improve interpretability by facilitating the identification of DIF and DSF items, quantifying effect sizes, and reducing tree complexity (Henninger et al., 2024, Preprint).

The $\left(p_{\gamma }\right)$ coefficient is a well-established descriptive measure for DIF and DSF in polytomous items, accompanied by a statistical significance test and a classification system, similar to the Mantel-Haenszel odds ratio for dichotomous items. Incorporating $\left(p_{\gamma }\right)$ into PCtree models provides a data-driven method to detect DIF and DSF, while enhancing interpretability through more concise trees and item-specific effect size quantification (Henninger et al., 2024, Preprint). As demonstrated in Table 1, Bjorner et al. (1998) presented a classification scheme for $\left(p_{\gamma }\right)$ which divides it into three categories: A for negligible effects, B for medium effects, and C for large DIF and DSF effects. This classification system enables researchers to assess the significance of item splits and quantify DIF and DSF effect sizes accordingly (Henninger et al., 2024, Preprint).

**Table 1.** Classification Rules for the Partial Gamma ( $p_{\gamma }$ ).

DIF Classification	Rule	$H_{0}$ Test
A ‒ negligible DIF/DSF	$\left|p_{\gamma }\right|$ ≤ 0.21	Or not significantly different from 0
B ‒ moderate DIF/DSF	Neither A nor C	And statistically significant
C ‒ large DIF/DSF	$\left|p_{\gamma }\right|$ ≥ 0.31	And significantly greater than |0.21|

### Previous Studies on Rasch Trees

Rasch trees are a relatively new technique that have seen limited use in educational and psychological research. A review of the literature identified only a handful of studies applying this method in these fields. Two lines of research can be identified in the relevant literature. Some of this research has applied the Rasch trees to investigate DIF of educational and psychological tests. For example, Aryadoust (2018) used recursive partitioning Rasch trees to detect DIF in a large-scale reading comprehension test across gender, grammar, and vocabulary. The analysis identified 11 distinct DIF groups, each showing significant variations in item difficulty. Results showed that DIF triggered by manifest variables only impacted specific subgroups with particular ability profiles, creating complex interactions between construct-relevant and -irrelevant factors. Yüksel et al. (2018) also investigated DIF of the Turkish version of the Nottingham Health Profile (NHP) across age, sex, and duration of pain. Using the mixed Rasch model, they first identified two latent classes. Then, using the Rasch tree method, it turned out that gender and age highly affected respondents’ item performance. In another study, Altıntaş and Kutlu (2020) investigated whether items in the Ankara University Examination for Foreign Students Basic Learning Skills Test show DIF across country and gender using Rasch tree. The results revealed DIF in 16 items at the 0.001 significance level, though these items exhibited similar difficulty parameters across countries. No DIF was found based on gender. Jeffers (2020) further explored DIF and item difficulty parameters of the Progress in International Reading Literacy Data (PIRLS) across multiple variables, both dichotomous and ordinal, and examined interactions between all variables included, without having to fit multiple models or define pre-set cut-points. The variables used in the recursive partitioning of the data were gender, a scale that measured attitudes toward reading (including enjoyment, motivation, confidence, and engagement), and correct or incorrect answers on a reading achievement scale, which was a 13-question scale based off a short story. The Rasch tree method effectively identified DIF and item difficulty for both dichotomous variables and interactions between dichotomous and ordinal variables. Hiller et al. (2023) evaluated the psychometric properties of the German version of the Overall Anxiety Severity and Impairment Scale (OASIS), a 5-item self-report measure that captures symptoms of anxiety and associated functional impairments. They used the RStree model to assess DIF across age and gender within both the total sample and the subsample of patients with panic disorder with/without agoraphobia. The analysis detected noninvariant subgroups associated with age and gender. Effatpanah et al. (2024c) recently used the RStree model to investigate DIF of the simplified version of the Beck Depression Inventory (BDI-S; Schmitt & Maes, 2000) across age and gender. The results showed that the interaction of gender and age affect depression manifestation, and the RStree could capture the underlying interaction between the covariates and the BDI-S items.

Some of this research has also endeavored to extend Rasch trees and develop new tree-based models by comparing them. For instance, Sarra et al. (2013) discussed the use of mixed-effects Rasch models for DIF analysis and proposed a unified framework that integrates the terminal nodes of the Rasch tree into a multilevel Rasch model to address various measurement issues. Using a cross-national survey on attitudes toward female stereotypes, the effectiveness of the approach was demonstrated. Ranger and Kuhn (2017) also developed a method to mitigate careless responding and identify unmotivated test takers in low-stakes tests. Drawing inspiration from the Rasch tree model, their approach segmented the data based on response times, allowing to isolate motivated test takers for improved model calibration. Unlike traditional Rasch trees that rely on data-driven splits, the method applied theoretically informed splits and selected optimal configurations using information criteria. Through a simulation study, the performance of the new method was evaluated against alternative models, including Meyer’s latent class model and a finite mixture model for response times. Their results showed that the method could effectively reduce bias associated with low motivation in specific scenarios. Furthermore, Bollmann et al. (2018) introduced an item-focused tree method for detecting DIF items that may impact the PCM. The approach generates a tree for each identified DIF item, visually highlighting which variables cause DIF and how they influence performance. The method was compared with Rasch tree PCM, and simulations indicated its more effectiveness. More recently, Tutz (2022) introduced new versions of ordinal trees and random forests that maintain the natural order of data without assigning artificial scores to categories. The method builds on binary models used in parametric ordinal regression, which are fitted as trees and combined like in parametric models. These trees strictly use the ordinal scale and incorporate existing binary trees and random forests. The method also addresses the often-overlooked issue of random forests underperforming in certain situations, suggesting ensemble methods that include parametric models for more accurate predictions across different datasets. Using several datasets, results showed the effectiveness of the methods. Taken together, all these empirical and simulation studies indicated the effectiveness of Rasch trees approach for detecting DIF and provide insights into how Rasch tree approaches can be integrated or compared with other models.

## The Present Study

The main purpose of this study is to demonstrate the usefulness of the RStree model (Komboz et al., 2018) in analyzing DIF of Likert scales in social sciences. To achieve this, the model is applied to the CTAS to identify causes of DIF. The covariates used in this study for recursively partitioning of the data are age and gender, as two most widely used covariates in DIF analyses. With respect to the above-mentioned previous studies indicating that respondents’ demographic variables can impact their performance on test anxiety scales, it is hypothesized that variations in gender and age may contribute to differential item performance among subgroups of respondents. More specifically, it is supposed that gender and age may induce DIF in the CTAS, and their interplay can impact the functioning of certain items within the scale and change the patterns of item difficulties across various subgroups. For the purpose of this illustrative study, the following research questions were addressed:

RQ1 Does the CTAS exhibit DIF toward respondents with regard to gender?

RQ2 Does the CTAS exhibit DIF toward respondents with regard to age?

RQ3 Does the interaction of age and gender change the pattern of item difficulties across subgroups?

RQ4 Can the RStree model capture the interaction between the covariates causing DIF?

## Method

### Data

Data analyzed in the present study included item responses of 721 Iranian EFL students to the Persian translation of the CTAS (Baghaei & Cassady, 2014). The age range of the students was 17–68 years (*M* = 22.40, standard deviation [*SD*] = 5.06), with Persian as their first language. There were 423 (58.7%) female and 298 (41.3%) male respondents. This relative gender disparity is due to the typical distribution of students in English departments in many Iranian universities. The data is part of a research project conducted by the author(s) to explore multiple profiles of test anxiety. The CTAS consists of 17 items scored on a four-point ordered response rating scale. The points on the scale are: 1 = not at all typical of me, 2 = somewhat typical of me, 3 = quite typical of me, and 4 = very typical of me (See Appendix A). No item required reverse scoring, and lower scores indicated lower levels of test anxiety. The Cronbach’s alpha reliability of the scale was 0.909, indicating satisfactory internal consistency of the scale. Informed consent was obtained from all individual participants included in the study. All participants were assured of the confidentiality of their responses.

### Data Analysis

#### Rating Scale Model Analysis

The *eRm* package version 1.0–6 (Mair et al., 2024) in the R statistical software (R Core Team, 2024) was used to analyze the data with the RSM (Andrich, 1978). The *eRm* package uses CML estimation for the dichotomous and polytomous Rasch models. The purpose of this preliminary analysis was twofold. First, the Rasch model should be initially fitted to the entire sample before using the RStree model to ensure that estimates of the latent trait are reliable and to make valid inferences about respondents’ abilities or item difficulties. Second, it was conducted to discover any anomalies in the data, diagnose rating scale structure, and detect potential sources of construct-irrelevant variance, which is a serious threat to test validity.

The Rasch model estimates item difficulty measures based on the proportion of respondents endorsing a specific item. Lower item difficulty values show that the item is highly endorsable and vice versa (Bond et al., 2020). For each item, an error of measurement index is also estimated to show the accuracy of the item difficulty parameters.

When the data deviate from the model, item difficulties and person estimates become inaccurate. To investigate the model-data fit, we computed two sorts of mean square (MNSQ) fit statistics (Linacre, 2024). The first sort of fit statistics was infit (MNSQ) which is an inlier pattern-sensitive fit index. This relies on the chi-square statistic, where each observation is weighted according to its statistical information (model variance). It is particularly attuned to detecting unexpected patterns in observations by persons on items that are roughly targeted on them (and vice-versa). The second sort was outfit MNSQ which is an outlier-sensitive fit index. This relies on the traditional chi-square statistic and is particularly attuned to detecting unexpected responses from persons on items that are either relatively easy or difficult for them (and vice versa). For polytomous data, a tolerated range for infit and outfit MNSQ values is 0.60–1.40 (Linacre, 2024). A MNSQ value of 1 is ideal fit. A value < 0.60 shows overfit and less variation than expected by the model. It is typically benign. However, a value > 1.40 indicates misfit and abnormal response patterns in comparison with the model’s expectations. This induces construct-irrelevant variance and distorts measurement (Baghaei & Effatpanah, 2022).

Smith and Plackner (2009) contend that infit and outftit MNSQ statistics are not very susceptible to systematic threats to unidimensionality. Consequently, using the *psych* package version 2.4.6.26 (Revelle, 2024) in R (R Core Team, 2024), the principal component analysis of linearized Rasch residuals (PCAR) was used to test the unidimensionality of the scale. Since items typically fail to conform to the expectations of the Rasch model, there are some remaining residuals after data-model fit. Residuals are differences between predictions of the Rasch model and the observed data. In fact, they are unexpected part of the data, which do not match the Rasch model. Residuals are expected to be uncorrelated and randomly distributed (Linacre, 2024), and when the values of residuals are smaller, the data better fit the model. When PCAR is carried out on the basis of standardized residuals, the latent trait is eliminated from the analysis; therefore, any component extracted from the residuals is taken as a second dimension, suggesting the violation of unidimensionality assumption (Linacre, 2024). The strength of the emergent component (e.g., the capacity of the component for describing the common variance in data) is compared with the strength of the target dimension. Linacre (2024) suggests that eigenvalues lower than 2 verify the unidimensionality of the test.

Moreover, a key characteristic of the Rasch model lies in its capacity to depict both the location of item and threshold parameters, alongside the distribution of person parameters, on a single latent trait scale. Person-item maps, also referred to as Wright maps, serve as valuable tools for comparing the relative locations and ranges of person and item measures.

The Rasch model also provides reliability coefficients for items and persons as well as separation statistics. Separation reliability denotes the ratio of true score variance to error score variance. It varies from zero to infinity and shows the degree to which person and item parameters are distinguishable on the latent trait (Linacre, 2024). Reliability spans from 0 to 1. Rasch person reliability indicates whether the ordering of respondents can be reproduced when they are given a set of equivalent items measuring the same latent trait. A reliability value > 0.7 is considered acceptable, and values above 0.8 are deemed favorable (Bond et al., 2020).

Finally, the functioning of thresholds between response categories of the scale was investigated using Rasch-Andrich (i.e., adjacent categories) thresholds. For items with multiple response options, there should be generally an association between trait levels and selecting response options, assuming they align in the same direction; choosing higher response categories typically corresponds to higher trait levels and vice versa. Consequently, estimated thresholds should increase alongside category values. When thresholds appear disordered, it suggests that “a category occupies a narrow interval on the latent variable or is poorly defined for respondents” (Linacre, 2024, p. 662). It must be noted that disordered Andrich thresholds do not violate Rasch models, but they can impact our interpretation of how the rating scale functions. Furthermore, ICCs which graphically represent the probability of endorsing a response category, contingent upon the position of individuals on the latent variable, were checked.

#### Rating Scale Tree Model Analysis

After checking the fit of the Rasch model to the entire sample, the RStree model (Komboz et al., 2018) was used to investigate DIF and DSF in the CTAS across gender and age using the *psychotree* package version 0.16–1 (Zeileis et al., 2024) in R (R Core Team, 2024). The null hypothesis of measurement invariance was that a single RSM holds for the entire sample. The hypothesis is rejected if the analysis produces multiple nodes, indicating that the RSM no longer adequately fits the data. Instead, there will be distinct item difficulty parameters for each subgroup of respondents, as defined by their group memberships or the covariates. The package visually presents the tree structure and the nodes.

The following consecutive steps were used to infer the structure of the RStree model: (1) using CML approach, item parameters were jointly estimated for all subjects across the entire sample; (2) the stability of the item parameters with respect to each covariate was evaluated; (3) in cases of significant parameter instability, the sample was split along the covariate with the most significant instability. Because the RSM requires a certain sample size to reliably estimate parameters for the items of the scale, the minimum sample size for terminal nodes was set to *N* = 250 (Debelak et al., 2022; Linacre, 2024); (4) after the subgroups resulting from the split were established, $\left(p_{\gamma }\right)$ was calculated for each item. Researchers can apply sum score purification and correct for item-wise testing using Bonferroni (Bonferroni, 1936) or false discovery rate (FDR; Benjamini & Hochberg, 1995) methods; and (5) Based on the $\left(p_{\gamma }\right)$ values, items were classified as having negligible, medium, or large DIF/DSF. If all items show negligible DIF/DSF, the tree split is undone to create more concise and interpretable trees (Henninger et al., 2024, Preprint). This allows researchers to evaluate which items exhibit DIF/DSF at each split and estimate the magnitude of these effects.

To evaluate the stability of a tree, we analyzed the stability of the splits using the *stablelearner* package version 0.1–5 (Philipp et al., 2023) in R (R Core Team, 2024). A single tree representation does not reveal which splits are stable, but this can be determined from an ensemble of trees generated by resampling the original data (Philipp et al., 2016). From the ensemble, the stability of the splits is assessed by examining the frequency of variable selection and the variability of cut-points across these resampled trees. Two steps are involved in this process (Philipp et al., 2016). First, multiple samples are drawn from the original dataset. Second, descriptive measures and graphical outputs are computed across all samples. The package offers several sampling methods, including bootstrap (sampling with replacement), subsampling (without replacement), k-fold sample splitting, leave-k-out jackknife sampling, and other user-defined strategies. In this study, we employed bootstrap sampling, the most common method, which is set as the default in the function *stabletree()*.

The goal of the variable selection analysis is to determine whether variables selected for splitting in the original tree are consistently selected in the resampled datasets. Additionally, the average frequency with which a variable is selected in the original tree versus the resampled trees is compared. Because even when the same variables are selected, splits can still differ in the chosen cut-points. Therefore, a crucial aspect of stability assessment involves analyzing cut-point variability, which offers deeper insights into the consistency of the splits (Philipp et al., 2016).

## Results

### Rating Scale Model Analysis

Table 2 shows the results of descriptive statistics, including mean, standard deviation (SD), skewness, and kurtosis, calculated on SPSS for Windows (Version 29), RSM item difficulty estimates, standard error (S.E.) associated with each item difficulty estimate, and item fit MNSQ statistics for the CTAS-17. The range of item means was from 1.58 to 2.58 and SD values from 0.744 to 1.003. Lower item mean indices represent lower endorsability of items, which are analogous to higher item difficulty measures. Low SD shows that data tend to cluster around the mean, and high SD indicates a high deviation from the mean. The skewness and kurtosis values also fell within the accepted range (±2), indicating that the data is normally distributed.

**Table 2.** Descriptive Statistics, Item Difficulties, and Fit Statistics for CTAS-17 and CTAS-16.

Items	Descriptive Statistics				CTAS-17				CTAS-16			
	Mean	SD	Skewness	Kurtosis	Measures	Model S.E.	Infit MNSQ	Outfit MNSQ	Measures	Model S.E.	Infit MNSQ	Outfit MNSQ
1	1.91	0.912	0.762	−0.259	0.194	0.053	1.190	1.215	0.134	0.054	1.252	1.268
2	2.13	0.744	0.542	0.344	−0.252	0.051	0.732	0.779	−0.338	0.052	0.783	0.837
3	1.92	0.882	0.738	−0.168	0.181	0.053	0.858	0.836	0.121	0.055	0.883	0.856
4	1.87	0.842	0.779	0.047	0.278	0.054	0.819	0.795	0.224	0.055	0.858	0.835
5	2.17	0.958	0.482	−0.680	−0.332	0.050	0.917	0.883	−0.423	0.052	0.946	0.909
6	1.96	0.963	0.681	−0.556	0.078	0.052	0.944	0.880	0.012	0.054	0.980	0.909
7	1.83	0.838	0.855	0.188	0.372	0.055	0.832	0.810	0.323	0.056	0.864	0.842
8	2.03	0.980	0.608	−0.665	−0.065	0.051	1.181	1.124	−0.140	0.053	1.253	1.202
9	2.45	0.882	0.209	−0.672	−0.848	0.050	0.834	0.873	−0.972	0.052	0.890	0.944
10	1.98	0.877	0.606	−0.356	0.055	0.052	0.931	0.904	−0.013	0.054	0.961	0.936
11	1.58	0.776	1.287	1.110	1.016	0.062	1.033	0.945	1.003	0.064	1.067	0.960
12	2.08	0.909	0.585	−0.392	−0.159	0.051	0.868	0.845	−0.240	0.053	0.905	0.892
13	1.90	0.764	0.769	0.617	0.214	0.053	0.804	0.860	0.156	0.055	0.863	0.917
14	2.01	0.753	0.496	0.106	−0.014	0.052	0.698	0.723	−0.086	0.053	0.749	0.787
15	2.58	1.003	−0.001	−1.088	−1.077	0.050	1.711*	1.928*	-	-	-	-
16	1.83	0.889	0.857	−0.092	0.378	0.055	1.032	0.973	0.330	0.056	1.093	1.027
17	2.01	0.887	0.602	−0.346	−0.020	0.052	0.941	0.918	−0.092	0.053	1.025	1.026

*Note.* *indicates misfitting item; SD = Standard Deviation; S.E. = Standard Error of Measurement; MNSQ = Mean Square.

The Rasch analysis of the CTAS-17 items showed a range of item difficulties spanning from −1.077 to 1.016 logits. Item 11 was the most difficult, and Item 15 was the easiest, suggesting that it was endorsed highly by the respondents. Person parameters also varied from −2.800 to 5.743. The values of infit and outfit MNSQ statistics showed that only Item 15 deviated from the desired range of 0.60–1.40, indicating anomalous response patterns that result in measurement distortion and represent cases of construct-irrelevant variance or multidimensionality. To construct a Rasch model-fitting measurement instrument, the item was omitted, and the fit of the data to the model was reanalyzed, leading to a shorter version of the scale (CTAS-16).

As depicted in the latter section of Table 2, the reanalysis of the CTAS-16 yielded results indicating that all items fell within the acceptable range, suggesting the absence of unexpected response patterns in the data. The Rasch person and item reliability indices were 0.89 and 0.99, respectively, implying that the ordering of persons and items is expected to be consistent across future studies.

Furthermore, PCAR was carried out to detect multidimensionality of the scale. As can be seen in Figure 1, the eigenvalue of the first factor (contrast) was slightly above 2, indicating the presence of an additional dimension and the lack of adherence of the responses to the Rasch model’s requirement of unidimensionality. This might be due to the presence of DIF.

**Figure 1.**
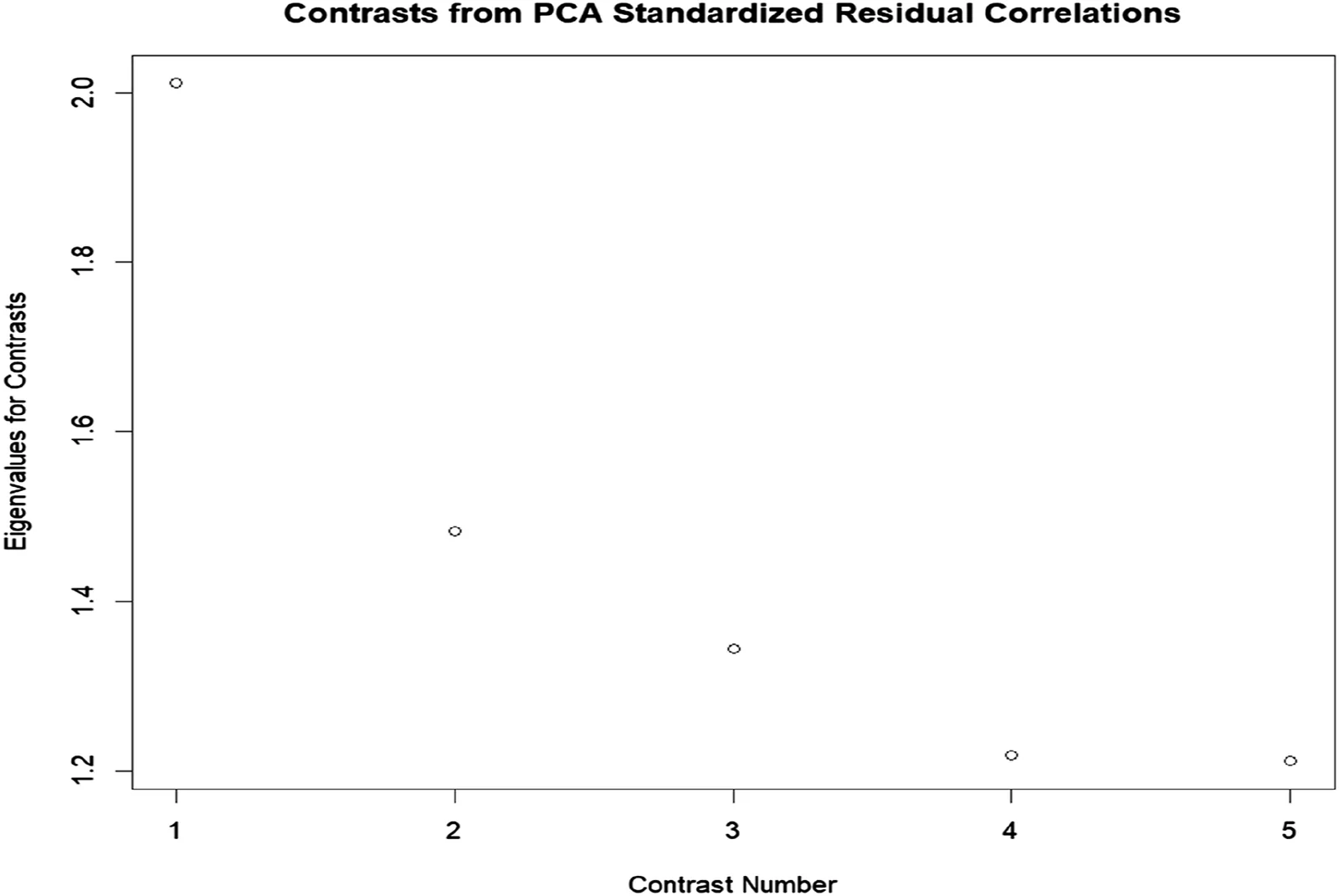
The results of five contrasts from PCAR.

Finally, the Andrich thresholds for items of the scale were analyzed. The threshold values were found to be at −1.664, 0.433, and 1.231, respectively, showing that the categories are correctly ordered and increase monotonically. As illustrated in Figure 2, the category probability curves for each item was also checked. The curves showed that all categories peak at certain points along the continuum and are logically ordered.

**Figure 2.**
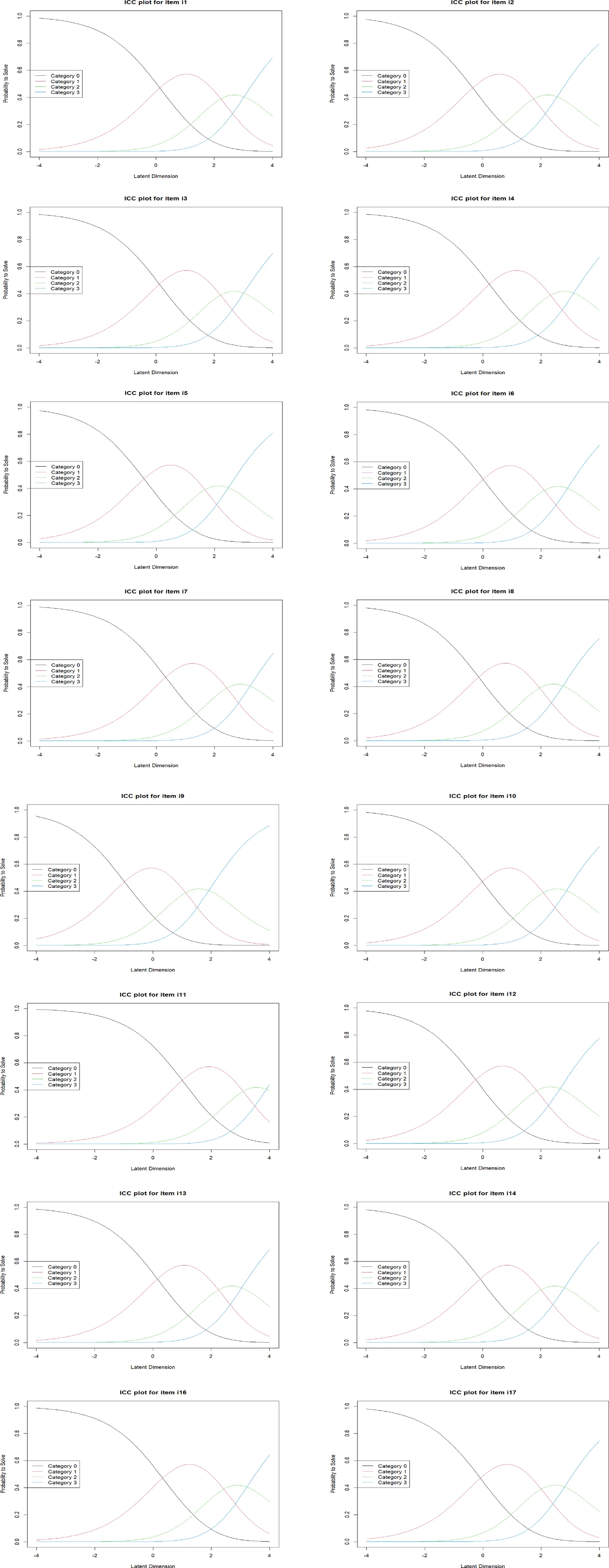
Rating scale category probability plots. *Note.* The overall shape of the curves and the relative distance between them is consistent across the items. However, the position of the curves on the logit scale differs across the individual items. The *eRm* package shifts responses such that the lowest category is 0.

The person-item map for RSM is demonstrated in Figure 3. At the bottom of the figure, the *x-*axis, tagged “Latent Dimension”, is the latent trait scale expressed in logits. Higher values indicate greater levels of test anxiety, while lower values indicate lower levels. The central panel of the figure illustrates item difficulty locations on the scale, and *y*-axis indicates the item labels. For each item, there is a solid circle plotting symbol which represents the overall item location estimate. The five open circles symbols denote the locations of the rating category thresholds. In the top part of the figure, a histogram of person location estimates on the scale is further shown. Small vertical lines on the horizontal axis of the histogram indicate points on the scale where variance is maximized for both the sample of items and persons in the analysis (Wind & Hua, 2022). The Wright map indicated that items cover a wide range of difficulty, providing evidence for the representativeness of the items. However, there is a mismatch at the extremes. On the lower end (below −1 logits), there are some persons with lower test anxiety, but fewer items are located here. Likewise, for persons with higher anxiety (above 4 logits), there is a gap as the scale lacks items to measure very high levels of test anxiety accurately. Therefore, adding more items that measure lower and higher levels of anxiety would improve the scale’s precision for this group.

**Figure 3.**
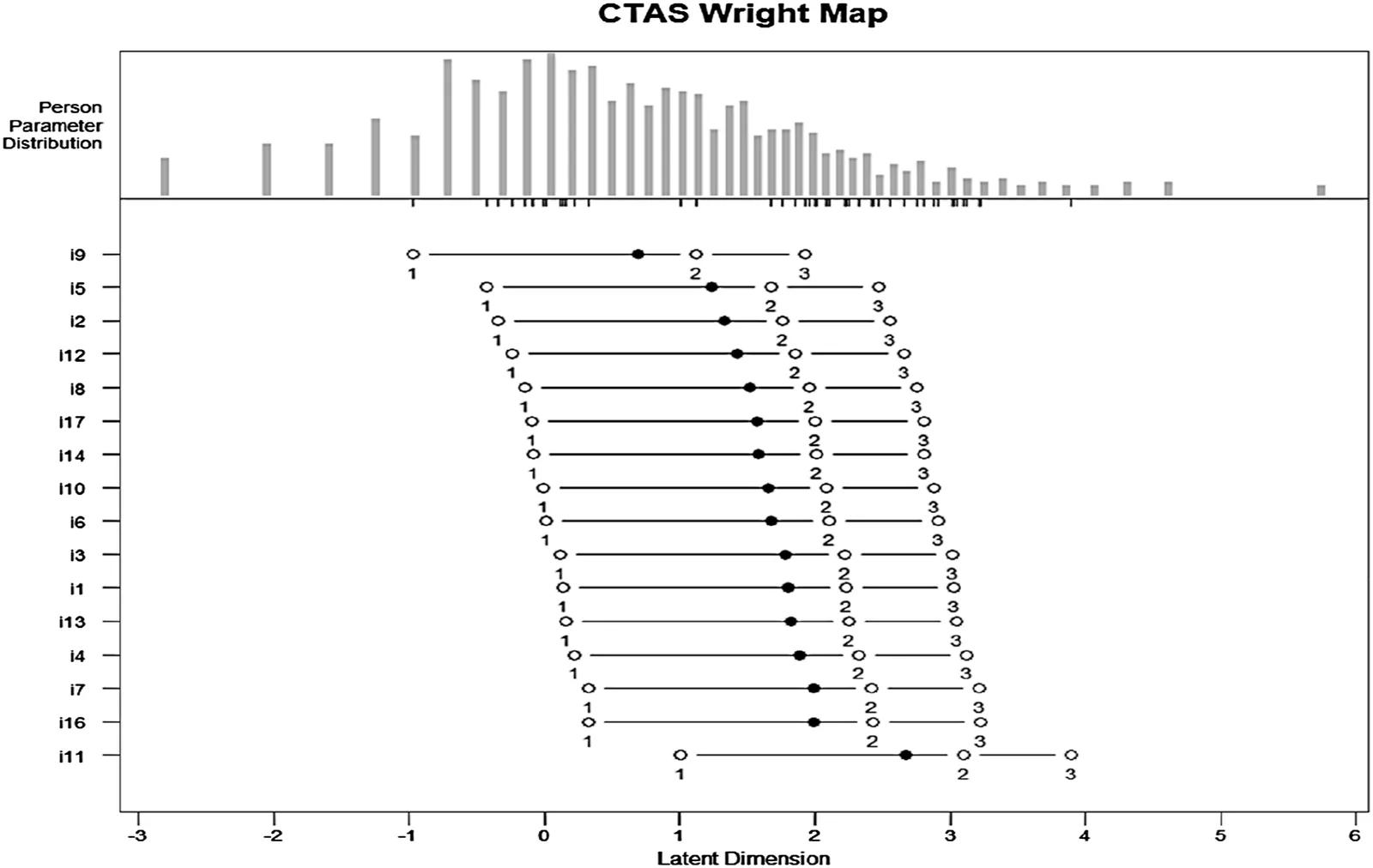
Person-item map of CTAS-16*. Note.* Items were sorted by their difficulties.

### Rating Scale Tree Model Analysis

To assess whether a single RSM is appropriate for all respondents using model-based recursive partitioning, the item responses were analyzed to detect potential group disparities caused by gender and age. As demonstrated in the item location profile plot (Figure 4(a)), there was more than one terminal node, suggesting the rejection of the null hypothesis of measurement invariance (i.e., a single RSM is applicable across the whole sample). The resulting model comprised 3 nodes, with two terminal nodes indicated by the rectangles at the lower part of the tree (Nodes 2 and 3), where item difficulty parameters for the 16 items of the scale are graphically displayed. Overall, no interaction between the age and gender variables was detected; only gender emerged as influential variable affecting test anxiety manifestation, and age did not have any impact on respondents’ item performance. The first node (*p* < .001) revealed gender-based differences in item difficulties, splitting the sample into two distinct subgroups. This indicates that gender significantly influenced response patterns, affecting how males and females responded to the CTAS. The first group (Node 2) consists of 298 males, and the second group (Node 3) includes 423 female respondents. This splitting pattern indicates variations in item difficulties across the subgroups, serving as empirical evidence that measurement invariance failed to hold among respondents from different subgroups, and they were differently impacted by DIF across the gender variable. The *x*-axis of the plots across the two terminal nodes shows the items, while the *y*-axis displays the range of item difficulties, spanning from −1.22 to 1.75 logits. The item difficulty patterns across the terminal nodes showed that Items 11 and 7 are more difficult for males, whereas Items 11 and 13 are more challenging for females.

**Figure 4.**
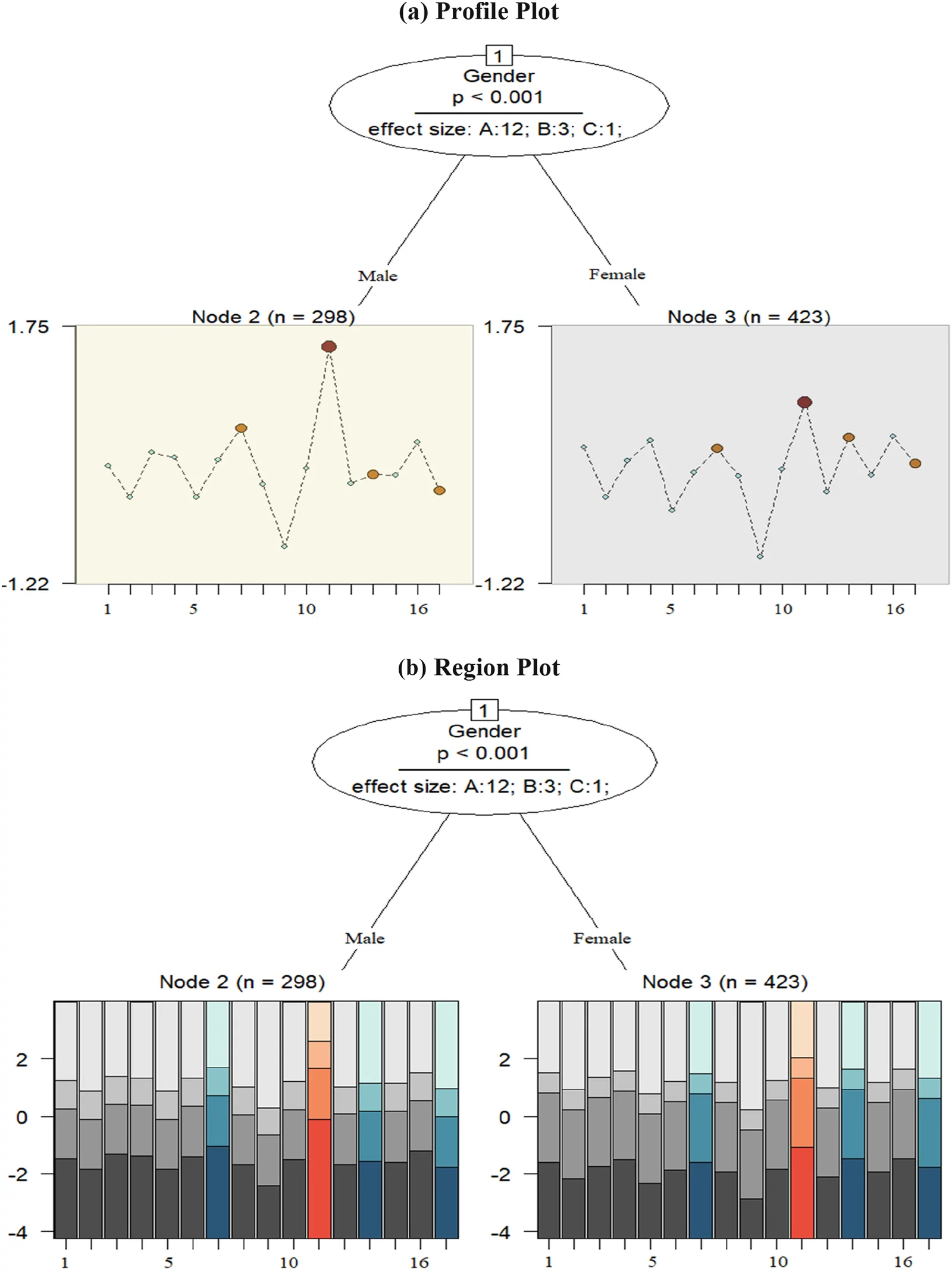
Region and profile plots of the RStree for the CTAS-16. (a) Profile Plot, (b) Region Plot. *Note.* Item 15 was removed. Circles represent variables associated with DIF.

Furthermore, effect sizes in the first node indicated that 12 items exhibited negligible or no DIF/ DSF (Category “A”), three items showed moderate DIF/DSF (Category “B”), and one item displayed large DIF/DSF (Category “C”) based on the covariate “gender.” In the profile plot, items with DIF/DSF are highlighted in red and orange based on their intensity, while DIF-free items are marked in blue. These DIF-free items, also known as anchor items, providing a stable reference for comparing item difficulty between gender-based subgroups (Glas & Verhelst, 1995). As can be seen, Items 7, 11, 13, and 17 showed DIF or DSF effects.

The region plots for each item in the terminal nodes are depicted in Figure 4(b). Regions of expected item responses are illustrated below each terminal node to display the respective item threshold parameters and check how individual category proportions are different between the subgroups identified by the RStree model. In fact, the plots visually depict the regions of the most probable category responses across the range of the latent trait (e.g., cognitive test anxiety) (Komboz et al., 2018). The regions are defined by threshold parameters estimated for the RSM within each node and can be used as a first descriptive evidence regarding specific items or categories affected by DIF. The *x*-axis of the plots across the two terminal nodes shows the items, while the *y*-axis displays the estimated threshold parameters of the RSM in the corresponding node, spanning from −4 to +4 logits. An inspection of the region plots showed that for both male and female respondents (Nodes 2 and 3), responses to the first and last categories, shaded in the darkest and lightest gray, were more probable, with slightly higher probability for males. More particularly, a higher latent trait was required for male and female respondents to choose the highest categories. However, males had a slightly higher probability for the third category, whereas responses to the second category was more probable for females. As can be seen, the region of Category 3 is narrower over all items for both female and male respondents. This can be can considered as an instance of DSF. Nevertheless, no disordered threshold parameters were observed across the terminal nodes. Threshold parameters for the terminal nodes are available in Appendix B.

As explained above, an important step in the RStree model is assessing the stability of the item or threshold parameters with respect to each available covariate. If there is significant instability, the algorithm splits the sample along the covariate with the strongest instability and in the cut-point leading to the highest improvement of model fit. The results of parameter instability tests with corresponding Bonferroni-adjusted *p*-values for the two covariates across all the nodes are presented in Table 3. Only the first node exhibited further data split, and no additional split points were chosen in the remaining nodes due to the lack of significant parameter instability warranting sample splitting (*p* > .05). For example, both age and gender exhibited significant *p*-values (<.05), but gender with the lowest *p*-value was chosen for the initial split in the first node. The split in Node 1 and the selection of the cut-point were straightforward owing to the binary nature of the gender variable, permitting only one split between male and female subgroups. No more appropriate covariates for splitting the data were identified within this node.

**Table 3.** Parameter Instability Tests Statistics and Their Associated Bonferroni-Adjusted p-Values.

Nodes	Age		Gender	
	Statistics	*p*-value	Statistics	*p*-value
Node 1	42.4509	0.012	1.26e + 02	5.39e-17*

*Note.* The variable whose *p*-value is highlighted with an asterisk was selected for splitting in the respective node.

Since the RSM is fitted within each node, we can observe how item difficulties differ between various subgroups, allowing us to detect items that function differently across covariates. If an item shows substantially different difficulty estimates across nodes, it suggests that respondents in those subgroups may interpret or respond to the item differently, beyond what is implied by their latent variable. The differences in item parameters are an indication of DIF which is formerly detected via splitting. Table 4 provides item difficulty estimates across the three nodes. Slight variations in item difficulties were observed across the nodes. The lowest and highest item difficulty values fell on Node 3 (i.e., −1.026) and Node 2 (i.e., 1.385), respectively. The most difficult item across the nodes was Item 11, whereas the easiest item in all nodes was Item 9. Such variations in item difficulty estimates across the three nodes reflect the differences in response behavior within each node. These differences occur because each node corresponds to a distinct subgroup of respondents, which might have distinct response tendencies, causing variations in item difficulty. Item 11 showed substantial difficulty differences across the three nodes (Node 1: 1.004, Node 2: 1.385, Node 3: 0.751), suggesting that respondents in Node 2 found the item much harder than those in Node 1 or Node 3. This item exhibited large DIF, as shown in Figure 4(a) and (b), and would be flagged for further investigation. Fluctuations in item difficulties across the three nodes are also illustrated in Figure 5.

**Table 4.** Item Difficulty Parameters Across the Three Nodes.

Items	Node 1	Node 2	Node 3
1	0.134	0.009	0.234
2	−0.338	−0.351	−0.336
3	0.121	0.165	0.083
4	0.224	0.107	0.318
5	−0.424	−0.351	−0.491
6	0.012	0.086	−0.052
7	0.323	0.452	0.222
8	−0.140	−0.201	−0.096
9	−0.972	−0.925	−1.026
10	−0.013	−0.019	−0.013
11	1.004	1.385	0.751
12	−0.240	−0.194	−0.284
13	0.156	−0.080	0.355
14	−0.086	−0.094	−0.085
16	0.330	0.283	0.367
17	−0.092	−0.273	0.054

*Note.* The highest item difficulty indices are underlined, and the lowest indices are emboldened.

**Figure 5.**
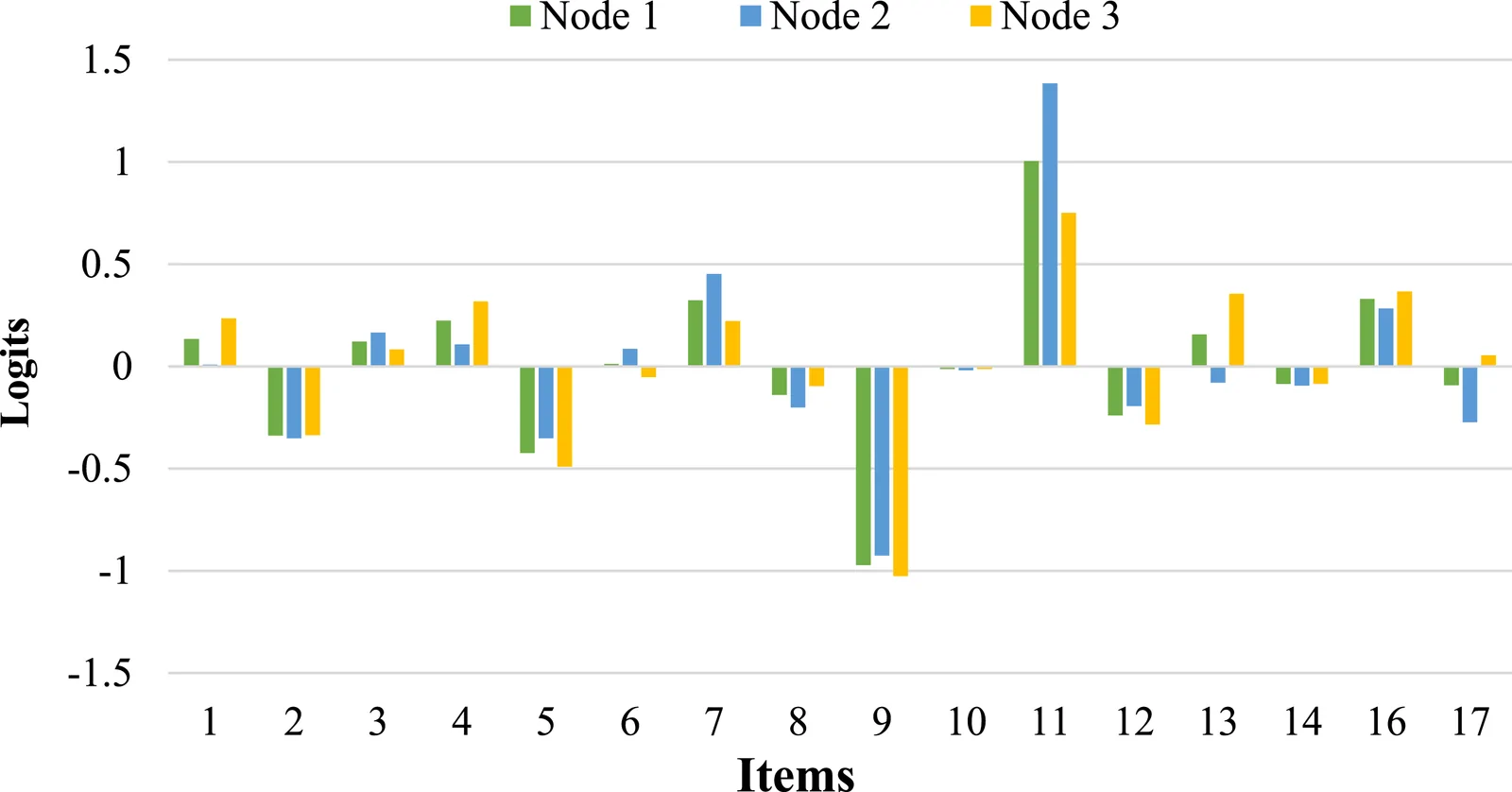
Patterns of item difficulty parameters across the terminal nodes.

### Stability Assessment

We investigated the stability of the splits to assess whether the tree is stable or not. Table 5 presents two key descriptive measures for variable selection analysis: the relative variable selection frequencies and the average number of splits for each variable (mean) across all 500 trees. By default, stabletree performs subsampling with a fraction of *v* = 0.632 of the original data and refits 500 trees. The relative frequency of a variable being selected for splitting is calculated across all repetitions of the procedure. If the result is stable and a variable consistently contributes to the model, the relative variable selection frequency should be high, ideally approaching 100%, for the variable selected in the original tree (Philipp et al., 2016). A relative selection frequency of 1 indicates that the variable was chosen in all 500 trees. The third column, marked with an asterisk, shows whether the variable was selected for partitioning in the original tree. The fourth column (labeled “mean”) provides the average number of splits for each variable per tree. This number can exceed one if a variable is used multiple times within a single tree. Ideally, the average number of splits should align with the number of times the variable was used for splitting in the original tree (Philipp et al., 2016). Finally, the last column, also marked with an asterisk, reflects the exact number of times the variable was used for splitting in the original tree. In this study, “gender” was chosen as a splitting variable once in every tree, including the original tree, whereas “age” was never selected for splitting in the tree.

**Table 5.** Variable Selection Overview.

	Frequency	*	Mean	*
Gender	1	1	1	1
Age	0	0	0	0

*Note.* *indicates original tree; All trees (B) = 500; Method = Subsampling with 63.2% data.

The frequency of variable selection can be graphically depicted in Figure 6(a). The variables along the *x*-axis are arranged in descending order based on their variable selection frequencies. The bars of variables selected in the original tree are highlighted in dark gray, with their corresponding labels underlined. The height of each bar reflects the corresponding variable’s selection frequency, as shown on the *y*-axis. As can be seen, the first bar reaches the maximum value of 100%, indicating that “gender” was selected for splitting in each iteration. However, the second bar, representing “age,” shows no selection, meaning it was not chosen in any of the repetitions. Moreover, the combinations of variables selected across different trees throughout the repetitions can be explored (Figure 6(b)). The repetitions, displayed along the *y*-axis, are arranged to group together similar combinations of selected variables. The specific combination of variables used for splitting in the original tree is highlighted on the right side of the plot with a thin solid red line, and the area representing this combination is enclosed by two dashed red lines. This combination is also the most frequently selected across all repetitions (Philipp et al., 2016). We observed again that “gender” was chosen as the splitting variable in all 500 trees. Overall, the variable selection analysis revealed that gender was consistently selected for partitioning, suggesting that it played a more significant role in affecting test anxiety manifestation compared to age.

**Figure 6.**
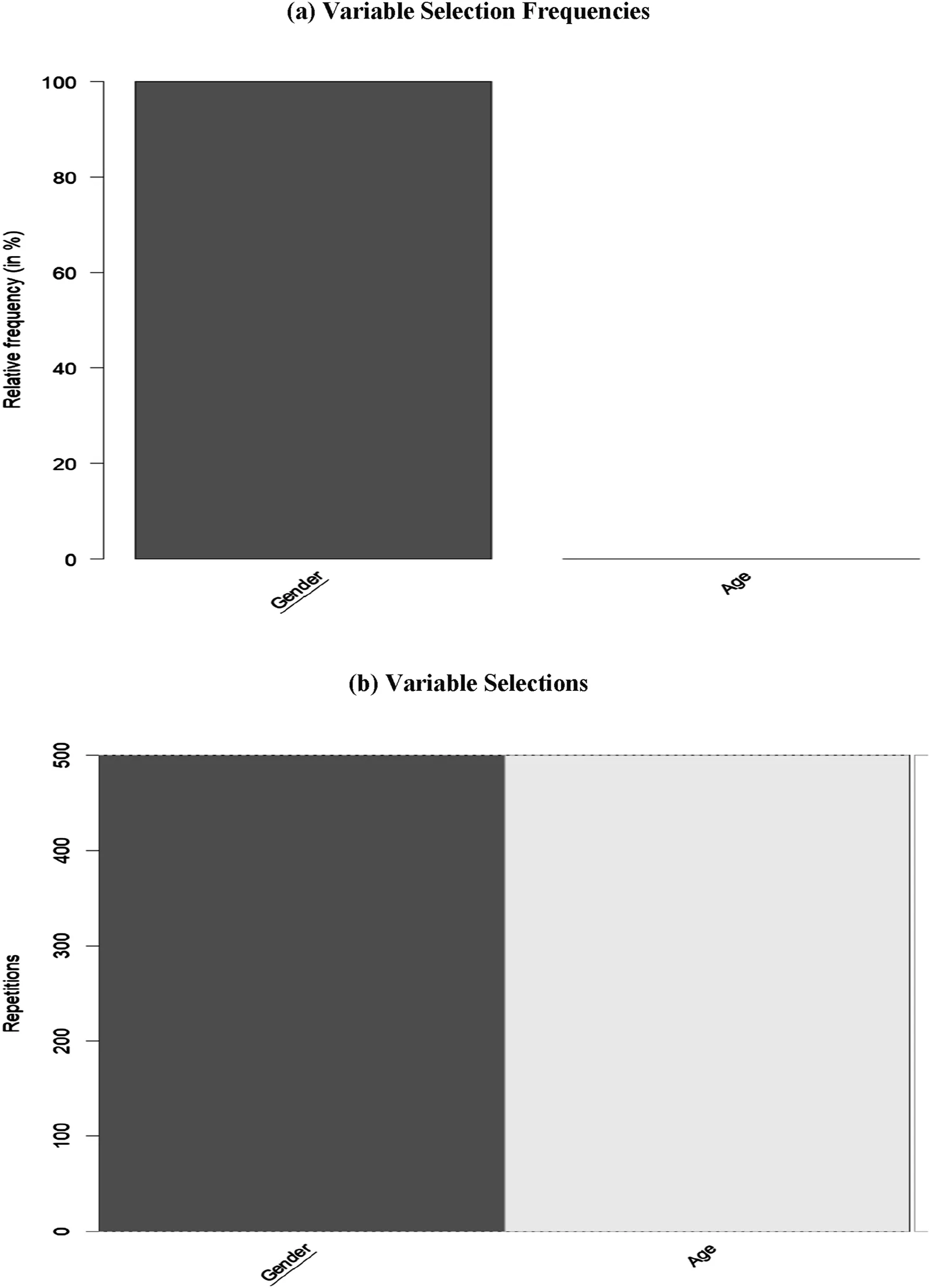
Graphical analysis of variable selection. (a) Variable Selection Frequencies, (b) Variable Selections.

Finally, as illustrated in Figure 7, we analyzed the variability of cut-points and the resulting partitions for each variable across all 500 trees using a barplot that displays the frequency of each possible cut-point. The cut-points are arranged along the *x*-axis according to the natural order of the variable’s categories. Variables selected in the original tree are indicated with underlined names (in this case, “gender”), and the cut-points chosen in the original tree are marked by vertical dashed red lines. The number above each red line denotes the specific level at which the split occurred in the original tree. For the binary variable “gender”, only one possible cut-point existed, which was used consistently whenever the variable was selected for splitting. This includes the first split in the original tree, as highlighted in red. Since “age” was not selected in the original tree, no corresponding plot was generated for it. Overall, the DIF effect for “gender” remained stable across all repetitions.

**Figure 7.**
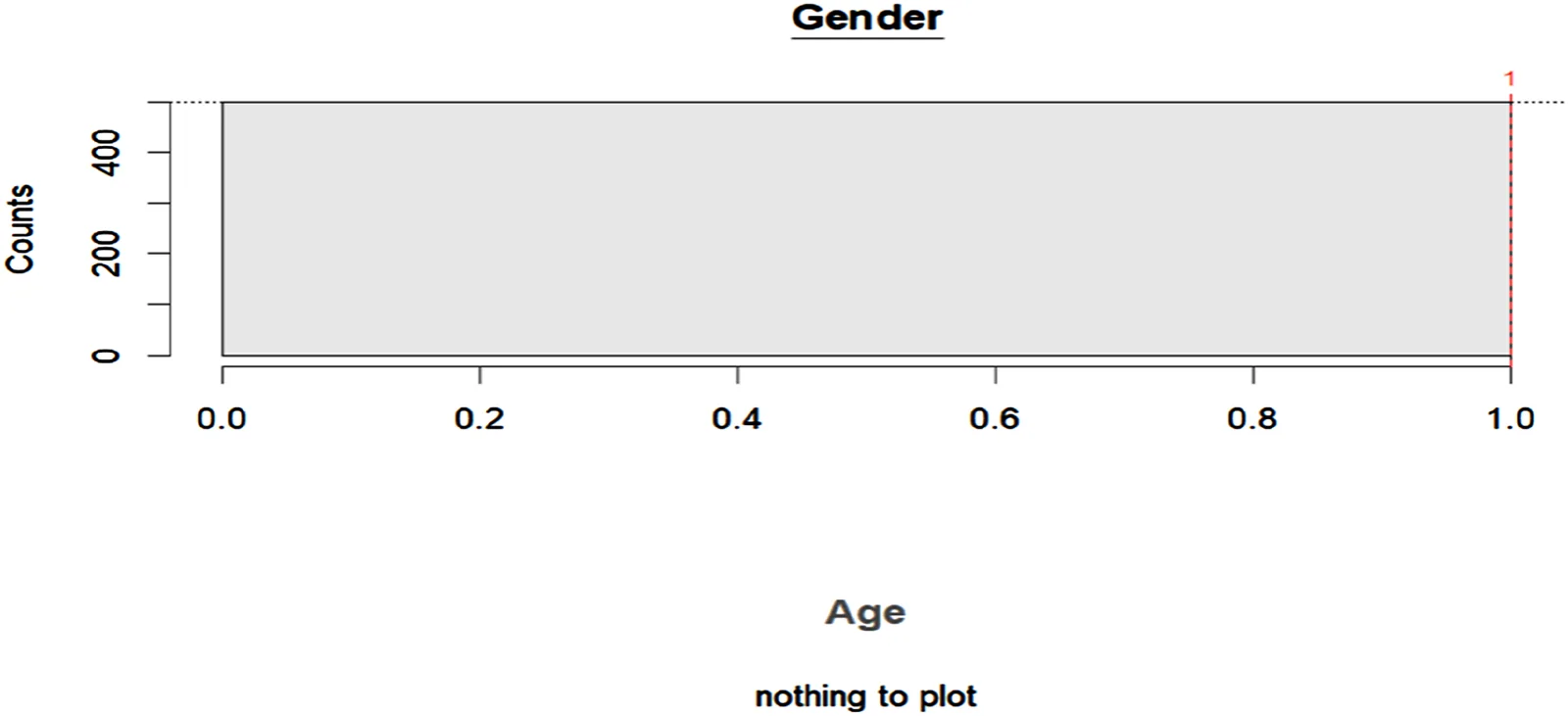
Graphical analysis of cut-points.

## Discussion

The present study aimed to apply the RStree model (Komboz et al., 2018) to a cognitive test anxiety dataset to demonstrate the usefulness of the model in detecting DIF of Likert-type scales across age and gender in social sciences. After assessing the compatibility of the data with the Rasch model, the RStree model was utilized to detect subgroups of respondents with distinct response patterns. Findings revealed the presence of three nodes and variations in item difficulty patterns among different respondent subgroups, indicating that a single RSM did not hold for the entire sample, that is, a single measurement model should not be used to compare the subgroups. The RStree model could also effectively represent the heterogeneity within subsamples. The respondents were only divided into two subgroups based on their gender (Node 1). The first group (Node 2) consisted of 298 male respondents, and the second group (Node 3) included 423 female respondents. This highlights variability in item difficulty estimates across respondents within the resultant nodes, contingent upon the gender as the partitioning covariate (Strobl et al., 2015).

An inspection of the tree structure and the nodes showed that age did not have any effect on the manifestation of test anxiety, whereas respondents’ item performance was affected only by the gender variable; males and females exhibited different response patterns to the CTAS items. To further investigate this, an independent-samples *t*-test was conducted to compare the total scores for males and females. The results showed that there is no significant difference between the scores for males (*M* = 34.23, *SD* = 9.139) and females (*M* = 34.22, *SD* = 9.805; t (719) = 0.013, *p* = 0.99, two-tailed). The mean difference was minimal (0.009), with 95% confidence intervals ranging from -1.406 to 1.425.The patterns of item difficulty, illustrated in Table 4 and Figure 4, indicated that there were some items where females exhibited higher item difficulty (i.e., Items 1, 2, 4, 8, 10, 13, 14, 16, and 17). However, the remaining items were more difficult for males (i.e., Items 3, 5, 6, 7, 9, 11, and 12), showing that they endorsed the items less. In the context of the cognitive test anxiety, higher item difficulty shows a lower probability of endorsing an item and lower level of test anxiety. In this study, for females, higher item difficulty is associated with lower levels of test anxiety. Essentially identical total scores suggest similar latent means, which contradicts previous research indicating that females, particularly adolescents, often report higher levels of test anxiety due to low self-efficacy and increased sensitivity to social approval (e.g., Torrano et al., 2020; von der Embse et al., 2018). There are some possible explanations for this contradictory result. First, females tend to exhibit more worry and emotionality—key components of test anxiety. However, the differences in how anxiety is expressed or experienced can vary by the type of anxiety assessed (e.g., cognitive, emotional). This study specifically focused on cognitive test anxiety, characterized by negative thoughts and performance-related concerns during exams (Cassady, 2023). Some studies suggest that males may report lower cognitive anxiety but experience greater physiological reactivity under testing pressure (Cassady & Johnson, 2002; Schmaus et al., 2008). This could explain why males in Node 2 found some items significantly easier than females (Node 3), suggesting higher levels of cognitive test anxiety that interfere with their test performance. Second, the slightly lower difficulty of some items for males may reflect social and cultural factors affecting how males and females address test anxiety. Males may experience significant cognitive disruptions during exams, leading to lower item difficulty estimates in the RStree analysis. As argued by Baghaei and Cassady (2014) in their investigation of DIF in the Persian version of the CTAS, societal pressures regarding job security may place greater expectations on males. For them, failure can delay graduation and diminish job prospects, whereas females may view failure as merely requiring them to repeat a course without similar career-related consequences. Third, it is possible that females have developed more effective coping strategies over time, particularly considering their generally higher self-reported anxiety levels in existing literature. This may explain why some females in this study found certain items easier to endorse, despite the overall trend indicating that females typically experience higher anxiety levels. Studies have shown that younger males have higher levels of test anxiety due to some factors, such as lack of experience in test-taking contexts and academic pressure (Putwain, 2007; Thomas et al., 2017; Torrano et al., 2020). As males mature, they might adopt more coping strategies to address test anxiety, reducing anxiety levels with increasing age.

Another debatable and contradictory finding of this study is the absence of DIF based on age. This contradictory result may be attributed to several possible reasons. First, the CTAS has been developed to measure various dimensions of test anxiety. However, if it is not detecting significant variations in test anxiety levels or experiences across different age groups, it may imply that the scale is not adequately effective for capturing the subtleties of how test anxiety manifests differently for students of varying ages. Further investigation is needed to explore this aspect of the scale in greater depth. Second, the sample used in this study consisted solely of undergraduate university students at a similar academic stage. Therefore, there may be relatively limited variation in the experience of test anxiety among different age groups. Research has indicated that test anxiety tends to be relatively stable in university settings, where students of different ages often share similar academic pressures, including expectations for performance, grading standards, and competition among peers, which can lead to a more uniform experience of test anxiety across age groups (Jerrim, 2022; Maier et al., 2021). Third, cognitive test anxiety scales typically address stressors that are relevant to all students, regardless of age (e.g., performance pressure, time constraints, etc.). In a university context, these stressors tend to resonate similarly with all students, leading to similar perceptions and reactions. Consequently, since all students face the same fundamental challenges related to academic performance, age may not significantly influence their responses to test anxiety items (Bonaccio & Reeve, 2010).

With respect to gender, the DIF effect sizes showed that there were four items exhibiting DIF; Item 11 was classified in Category “B” (large DIF), and three items (i.e., Items 7, 13, and 17) were classified as Category “A” (negligible DIF). A content analysis of the items was conducted to identify the causes of DIF. For example, Item 11 (i.e., During tests, I have the feeling that I am not doing well.) measures test-related self-efficacy and performance anxiety. Research shows that gender differences in self-assessment of performance can significantly affect responses to such items (e.g., Harris et al., 2019). Studies have found that females often report lower self-confidence and higher levels of self-doubt in academic settings compared to males, even when performance does not differ significantly (e.g., McMurran et al., 2023). This difference could explain why this item showed a large DIF effect, as females might be more likely to internalize test anxiety and perceive themselves as “not doing well or underperforming” compared to men, despite achieving similar outcomes to their male counterparts (Harris et al., 2019).

 Item 7 (i.e., During tests, the thought frequently occurs to me that I may not be too bright.) shows the feelings of inadequacy during tests. Previous studies indicated that gender stereotypes may increase the intellectual insecurity of women, causing an increase in their self-doubt in academic settings (Ertl et al., 2017). Consequently, women may be more inclined to experience thoughts of inadequacy during exams, questioning their intellectual abilities. As illustrated in Table 4 and Figure 4, the item difficulty for females (i.e., 0.222) was lower than for males (i.e., 0.452), showing a higher endorsement for females compared to their male counterparts because females are more likely to experience intellectual insecurity. Research also reported that women are more likely to report lower self-confidence in academic settings (Harris et al., 2019). This could explain why women found this item easier to agree with, as societal pressures often lead to more frequent and accessible thoughts of self-doubt for females during tests. By contrast, males may experience fewer doubts about their intelligence in test settings, or at least may be less likely to admit to such thoughts, possibly due to societal norms that discourage vulnerability in men, especially regarding intellectual performance. This could explain why males found this item more difficult to endorse, contributing to the moderate DIF effect observed between genders.

 Item 13 (i.e., I am a poor test taker in the sense that my performance on a test does not reflect how much I truly know about a subject.) shows a disagreement between test performance and the real knowledge of a student. Generally, women are more likely to face this issue due to the effect of stereotype threat (i.e., a belief stating that women are not competent enough in academic contexts) (Burgess et al., 2012). Stereotype threat can diminish performance, particularly in high-stakes environments, leading women to view themselves as less effective test takers (Sebastián-Tirado et al., 2023). The moderate DIF effect observed for this item may result from gender-based differences in how men and women perceive or experience the anxiety surrounding test performance.

 Item 17 (i.e., When I take a test, my nervousness causes me to make careless errors.) indicates an association between test performance errors and test anxiety. Because research has consistently reported a higher level of test anxiety among females, it is more probable that they will endorse this item (e.g., Torrano et al., 2020; Von der Embse et al., 2018). Females often experience greater test-related nervousness that leads to an increase in perception of making careless errors. The moderate DIF effect observed for this item likely stems from these gender-related variations in anxiety.

The examination of region plots, displayed in Figure 4, showed narrower regions for Category 3 in Node 3 (females) compared to males, indicating that females were less likely to endorse this moderate level of agreement. However, it must be noted that this observed narrowing could partially reflect the influence of fixed tau parameters within the model. Alternatively, this narrower region may reflect females’ difficulty in distinguishing between the highest response options on the CTAS. It could also be due to response style differences, where females might tend to express moderate or lower levels of agreement, while males show greater differentiation between moderate and higher levels (Categories 3 and 4). However, Category 2 in Node 3 is more prominent for females than for males, suggesting that females are more inclined to select this option, possibly due to uncertainty or difficulty in distinguishing between moderate and higher test anxiety responses. This may also indicate a tendency to “play it safe” or underreport higher levels of anxiety, opting for more neutral responses. Previous research has suggested that females may underestimate their abilities or experience greater self-doubt in testing situations (e.g., McMurran et al., 2023), leading to more frequent selection of mid-range options. While the region plots highlighted the presence of DSF, they did not indicate disordered thresholds, suggesting that the response categories were used in the intended order, despite the differing frequency of use between genders.

When considering the methodological aspects of the RStree model, a distinctive characteristic of the approach, compared to the conventional DIF detection methods, is the absence of necessity to prespecify groups for detecting DIF a priori. However, the process of forming DIF subgroups and determining cut-points in the model entails combining covariates (e.g., age and gender). As argued by Strobl et al. (2015, p. 293),

This is a key feature of the model-based recursive partitioning approach employed here, which makes it very flexible for detecting groups with DIF and distinguishes it from parametric regression models, where only those main effects and interactions that are explicitly included in the specification of the model are considered.

The model also offers a reliable statistical approach to examine invariance at the step level in polytomous items. Research has indicated that neglecting the impact of DSF leads to biased parameter estimates and reduces the effectiveness of conventional methods for detecting DIF (Finch, 2022).

Before wrapping up this section, as argued by Philipp et al. (2016), recursive partitioning techniques, such as CART, conditional inference trees, and model-based recursive partitioning, are commonly employed to explore and model unidentified structures within complex, potentially high-dimensional datasets. These methods excel at identifying nonlinear structures and complex interactions in a data-driven manner by recursively dividing the predictor space into homogeneous observation groups. Consequently, recursive partitioning has gained widespread use in predictive modeling across numerous scientific fields and industries (see Kuhn & Johnson, 2013 for a comprehensive review). While more advanced and flexible techniques, such as support vector machines, boosting, neural networks, random forests typically deliver superior predictive accuracy, tree-based recursive methods like the Rasch tree remain popular due to their ability to present results as decision trees, which are relatively straightforward to interpret (Strobl et al., 2009). However, these methods are limited in the following ways (Philipp et al., 2016): (1) they are instable in the sense that small variations in the data can influence the selection of split variables and the determination of cut-points, causing the resulting tree to take on a substantially different form; and (2) they do not provide confidence measures for the chosen splits, leaving users unable to evaluate the reliability of the selected variables, cut-points, or the overall tree structure. This raises concerns about the extent to which conclusions can be drawn from a single tree. To address these challenges, Philipp et al. (2016) developed a toolkit of descriptive measures and graphical visualizations based on resampling techniques that enable researchers to assess the stability of variable and cut-point selections in recursive partitioning. In this study, we employed bootstrap sampling to evaluate the stability of the splits. The findings indicated that gender was consistently selected for partitioning, indicating its important role in influencing the manifestation of test anxiety.

### Implications of the Study

This study offers several implications from different perspectives. From a methodological perspective, the results of this study demonstrate the usefulness of the RStree model in analyzing DIF of Likert-type scales in social sciences. By providing an empirical application of the RStree to the CTAS, it turned out that researchers can use the model to identify subgroups and their related covariates. Additionally, the utilization of the RStree model introduces a novel analytical methodology in social sciences research. This highlights the employment of sophisticated statistical techniques to explore intricate patterns and relationships within complex datasets, which can spark further methodological progress within social sciences.

From a theoretical perspective, the present study advances and broadens previous research on the use of statistical methods to detect DIF in social sciences. The utilization of the RStree model in analyzing DIF of Likert-type scales contributes to the development of measurement theory by offering a flexible framework that incorporates respondent-specific characteristics (i.e., covariates) into item response models. This approach allows for identifying complex DIF patterns by revealing how different subgroups of respondents may interact with test items in distinct ways. Specifically, it moves beyond traditional models with a priori fixed focal and reference groups by accommodating heterogeneity in item functioning that traditional models may overlook, thereby refining our understanding of latent trait measurement in complex, real-world settings. The results of this study highlight the importance of analyzing the interaction of test items and several covariates which affect the performance of respondents. The identification of item functioning patterns offers more detailed information about the nature of a latent trait, its manifestations, and its interaction with other related variables. Accordingly, this enhances researchers’ appreciation of the expected latent trait and facilitates the refinement or formulation of theories and models pertaining to the trait. In addition to gender and age, covariates may be selected based on theoretical considerations, prior empirical findings, or exploratory data analysis. For instance, factors such as educational background, test-taking experience, socioeconomic status, or psychological traits (e.g., anxiety or motivation levels) could be tested for their influence on item responses. These covariates can be chosen by conducting preliminary analyses, including variable selection procedures (e.g., stepwise regression, random forest variable importance) or through theory-driven hypotheses about variables expected to impact the latent trait in question. Since the tree-based methods employed here control the false positive rate, one can consider to err on the side of including more covariates than less, since an uninformative variable will not be used for splitting.”

From a practical perspective, the findings of this study emphasize the significance of constant improvement of Likert-type scales used in social sciences, especially the CTAS. Flagging items that function differently across subgroups can help researchers to improve the psychometric properties of scales.

### Limitations and Directions for Future Research

This study has several limitations that need to be addressed in future research. First, the applicability of the present results on the CTAS is limited to the current sample under investigation. Much more research is needed to investigate the effectiveness of the RStree model in detecting DIF of Likert-type scales in social sciences in various populations. Variations in demographics, language proficiency, and educational systems/levels could influence the outcome (Cassady & Johnson, 2002). Second, the study only incorporated two covariates to assess subgroup invariance. Future studies can expand the scope by including a variety of covariates, such as race/ethnicity, educational level, socioeconomic status, personality traits, etc., to identify potential sources of DIF using the RStree model. This expansion would provide a more comprehensive understanding of potential sources of DIF when using the RStree model in diverse populations. However, bear in mind that incorporating numerous covariates for DIF detection may lead to multiple testing issues. However, the trees use a Bonferroni-correction in each split to control for the effects of multiple testing (rather conservatively than liberal). By employing a closed testing procedure, the trees ensure that multiple splits do not inflate the Type I error rate, maintaining the overall significance level for the entire tree structure (Hochberg & Tamhane, 1987; Strobl et al., 2015). Future studies can also conduct post-hoc analysis to exactly identify those respondents who are affected by covariates.

A significant consideration in the application of tree-based models, such as the RStree model, is ensuring an adequate sample size to achieve sufficient statistical power for detecting DIF. The present study used a moderate sample size to demonstrate the RStree model. It may present limitations in the context of detecting DIF within the RStree framework. As noted earlier, tree-based models use recursive partitioning techniques to split data into subgroups based on covariates, in this case age and gender. To obtain reliable estimates and draw conclusions about DIF, each subgroup must contain sufficient respondents. Insufficient or smaller sample sizes can destabilize parameter estimates, increasing the risk of Type I or Type II errors. For example, with a limited number of responses, certain response patterns may not be adequately represented, leading to misleading conclusions about the presence or absence of DIF. Moreover, the nature of Likert-type scales introduces additional complexities, as response patterns often vary subtly across demographic groups. A larger sample size would improve the model’s ability to accurately capture these patterns and provide more accurate insights into potential DIF across age and gender. Larger samples would also support more detailed subgroup analyses, ensuring findings are more generalizable to broader populations. To address this limitation, future studies should incorporate larger and more diverse samples. This would strengthen the statistical power of the RStree model and provide a deeper understanding of how DIF exhibit across various demographic groups. With larger datasets, researchers could also capture interaction effects and investigate more complex relationships between demographic variables and response patterns.

As a reviewer of an earlier draft of this paper rightly pointed out, like most unsupervised learning models, an inherent weakness of the tree-based IRT models is model overfitting. Similar to other recursive partitioning models, the RStree model can be liable to overfitting due to its nature of partitioning data into smaller subgroups to improve model fit. However, several countermeasures mitigate overfitting in the RStree model. First, as mentioned, multiplicity adjustments such as Bonferroni correction or FDR control can be used when conducting multiple tests to reduce the likelihood of overfitting due to spurious findings. These adjustments help control the family-wise error rate, warranting that the splits (i.e., DIF detection) identified by the tree model are statistically meaningful rather than the result of chance. Second, tree pruning techniques can be used, which involve cutting down branches of the tree that do not significantly improve the model’s performance. Third, although regularization is more commonly associated with parametric models, there are similar methods for tree-based models. Penalizing splits based on the complexity or the depth of the tree can prevent overfitting by discouraging excessive partitioning. Fourth, cross-validation is a widely used approach for alleviating overfitting by training the model on one subset of the data and validating it on another. This allows for assessment of the model’s generalizability. Finally, as part of the model development process, researchers should not only rely on the statistical fit of the splits but also assess whether these splits make substantive sense with regard to theory and practical significance. 

Given that psychological constructs are likely to change over time, various factors might influence the consistency of item functioning within scales. Therefore, a vibrant line of research would be conducting longitudinal studies to illuminate the stability of item functioning across time. For that purpose, future studies could include the variable time (measured either numerically or at multiple discrete time points) for splitting the dataset, akin to other covariates. The identification of one or more splits in the model would indicate fluctuations in the item parameter estimates over time (Komboz et al., 2018).

Another area warranting further exploration involves the application of newly developed advanced tree models for investigating DIF. Numerous researchers have developed structural equation modeling (SEM) trees (e.g., Arnold et al., 2021; Brandmaier et al., 2013; Grassi & Tarantino, 2023; Lou et al., 2022) for cause-effect research contexts. The models conflate the strengths of SEMs and the decision tree paradigm by generating tree structures that recursively partition a dataset into subsets exhibiting remarkably different parameter estimates within a SEM framework (Grassi & Tarantino, 2023). Of particular interest is also the application of item-focused trees within the context of IRT for investigating DIF. Such models aid researchers in extracting item specific information (Bollmann et al., 2018; Berger & Tutz, 2016; Tutz & Berger, 2015). The application of such sophisticated methods will advance empirical investigation of DIF and optimize the accuracy of analyses in social sciences. Finally, future studies can use generalized partial credit tree model (De Boeck & Partchev, 2012) to include item discrimination parameters, which would provide a more intricate understanding of item performance across groups. This could enhance the analysis of DIF, particularly for polytomous items like those in Likert-type scales.

## Conclusion

This study set out to showcase the effectiveness of the RStree model in detecting DIF of Likert-type scales in social sciences. The advantage of the model is that unlike the conventional DIF detection methods, it does not require a priori specification of groups for investigating DIF, and numeric variables, such as age, do not need to be categorized before testing, which maintains valuable information in the analysis. To demonstrate the effectiveness of the model in analyzing DIF of Likert-type scales in social sciences, item responses of 721 EFL students to a cognitive test anxiety scale was analyzed. Age and gender were selected as covariates. Four items of the scale exhibited DIF. It turned out that age did not affect the item performance of respondents, while gender had a role in generating DIF in test anxiety. The results also indicated the effectiveness of the model in capturing the underlying interaction between the covariates and the scale items.

## Data Availability

The datasets generated during and/or analyzed during the current study are available from the corresponding author on reasonable requests.

## Appendix

### Abbreviations

CART: Classification and Regression Tree
CFA: Confirmatory Factor Analysis
CML: Conditional Maximum Likelihood
CTAS: Cognitive Test Anxiety Scale
CTT: Classical Test Theory
DIF: Differential Item Functioning
DSF: Differential Step Functioning
EFL: English as a Foreign Language
FDR: False Discovery Rate
ICCs: Item Characteristic Curves
IRT: Item Response Theory
LM: Lagrange-Multiplier
LR Test: Likelihood Ratio Test
M: Mean
MH: Mantel-Haenszel
MNSQ: Mean Square
NAEP: the National Assessment of Educational Progress
NHP: Nottingham Health Profile (NHP)
OASIS: Overall Anxiety Severity and Impairment Scale
PCAR: Principal Component Analysis of Linearized Rasch Residuals
PCM: Partial Credit Model
PCtree: Partial Credit Tree
PIRLS: the Progress in International Reading Literacy Data
PISA: the Programme for International Student Assessment
RSM: Rating Scale Model
RStree: Rating Scale Tree
SD: Standard Deviation
S.E.: Standard Error
SEM: Structural Equation Modeling
TIMSS: the Trends in International Mathematics and Science Study

## Appendix A: The 17-Item Revised English Version of the Cognitive Test Anxiety Scale (CTAS-17)

Please complete the following items using the four-point scale below: (1) = Not at all typical of me, (2) = Somewhat typical of me, (3) = Quite typical of me, (4) = Very typical of me.

**Table:**

Items	Content	Categories			
1	I lose sleep over worrying about examinations	1	2	3	4
2	While taking an important examination, I find myself wondering whether the other students are doing better than I am	1	2	3	4
3	I tend to freeze up on things like intelligence tests and final exams.	1	2	3	4
4	During tests, I find myself thinking of the consequences of failing.	1	2	3	4
5	At the beginning of a test, I am so nervous that I often can’t think straight.	1	2	3	4
6	My mind goes blank when I am pressured for an answer on a test.	1	2	3	4
7	During tests, the thought frequently occurs to me that I may not be too bright.	1	2	3	4
8	During a course examination, I get so nervous that I forget facts I really know.	1	2	3	4
9	After taking a test, I feel I could have done better than I actually did.	1	2	3	4
10	I worry more about doing well on tests than I should.	1	2	3	4
11	During tests, I have the feeling that I am not doing well.	1	2	3	4
12	When I take a test that is difficult, I feel defeated before I even start.	1	2	3	4
13	I am a poor test taker in the sense that my performance on a test does not show how much I really know about a topic.	1	2	3	4
14	I am not good at taking tests.	1	2	3	4
15	When I first get my copy of a test, it takes me a while to calm down to the point where I can begin to think straight.	1	2	3	4
16	I do not perform well on tests.	1	2	3	4
17	When I take a test, my nervousness causes me to make careless errors.	1	2	3	4

## Appendix B: Threshold Parameters for the Three Nodes

**Table:**

Items	Thresholds	Node 1	Node 2	Node 3
1	Threshold 1	−1.530	−1.482	−1.590
	Threshold 2	0.567	0.271	0.798
	Threshold 3	1.365	1.238	1.494
2	Threshold 1	−2.002	−1.842	−2.161
	Threshold 2	0.094	−0.088	0.228
	Threshold 3	0.893	0.878	0.924
3	Threshold 1	−1.542	−1.326	−1.742
	Threshold 2	0.554	0.427	0.647
	Threshold 3	1.352	1.394	1.343
4	Threshold 1	−1.439	−1.384	−1.506
	Threshold 2	0.657	0.370	0.882
	Threshold 3	1.455	1.336	1.579
5	Threshold 1	−2.087	−1.842	−2.315
	Threshold 2	0.009	−0.088	0.074
	Threshold 3	0.808	0.878	0.769
6	Threshold 1	−1.652	−1.405	−1.876
	Threshold 2	0.444	0.349	0.512
	Threshold 3	1.243	1.315	1.208
7	Threshold 1	−1.340	−1.040	−1.602
	Threshold 2	0.756	0.714	0.787
	Threshold 3	1.554	1.681	1.482
8	Threshold 1	−1.803	−1.692	−1.920
	Threshold 2	0.293	0.062	0.468
	Threshold 3	1.091	1.028	1.164
9	Threshold 1	−2.636	−2.416	−2.850
	Threshold 2	−0.540	−0.662	−0.462
	Threshold 3	0.259	0.304	0.234
10	Threshold 1	−1.676	−1.510	−1.838
	Threshold 2	0.420	0.244	0.551
	Threshold 3	1.218	1.210	1.247
11	Threshold 1	−0.660	−0.106	−1.073
	Threshold 2	1.436	1.648	1.315
	Threshold 3	2.235	2.614	2.011
12	Threshold 1	−1.904	−1.685	−2.108
	Threshold 2	0.193	0.068	0.281
	Threshold 3	0.991	1.034	0.976
13	Threshold 1	−1.507	−1.571	−1.470
	Threshold 2	0.589	0.182	0.919
	Threshold 3	1.387	1.148	1.614
14	Threshold 1	−1.749	−1.585	−1.909
	Threshold 2	0.347	0.169	0.479
	Threshold 3	1.145	1.135	1.175
16	Threshold 1	−1.333	−1.209	−1.457
	Threshold 2	0.763	0.545	0.931
	Threshold 3	1.561	1.511	1.627
17	Threshold 1	−1.755	−1.764	−1.770
	Threshold 2	0.341	−0.011	0.619
	Threshold 3	1.139	0.956	1.314

*Note.* Item 15 was removed.
